# General Index to the British and Foreign Medical Review

**Published:** 1848

**Authors:** 


Life, Dr. Paine on . . . xi 385
Dr. Paine's original views on . xv 144
duration of, an error respecting xvii 403
in different classes . xxi 10
effects of civilization on xviii 237, 246
Mr. Chadwick on . . xviii 197
tallying in families . . ii 6
that of peers . . . xxi 1, 9
effect of, on chemical action . xxi 493
expectation of, in medical men i 202
human, on the renewal of . xvi 208
Hunter on . . . . . v 47
in accordance with nature . xviii 237
in cells and organisms . . xx 71
increased duration of . . vi 196
in the sick-room . . . xviii 472
influence of temperature on . xix 182
insect, Dr. Badham on . . xxi 494
mean term of, at Geneva . i 194
medical, moral aspects of . xxiii 467
Mr. Carpenter on . . . vii 171
mode of, according to nature xviii 237, 245
Mr. Macilwain . . . vi 100
observations on . . . xiv 521
of the blood, Harvey's views of i 388
Hunter on the . . viii 178
of a travelling physician . . xvi 265
on its division into epochs . i 323
organic and animal, Dr. S. Smith on i 316
origin of . . . . . xix 169
physical phenomena of . . viii 309
probable duration of, in England xviii 200
Reil on . . . . . v 78
remark of Andral on . . viii 179
sedentary, its effects on longevity i 198
study of physical phenomena of viii 352
term of, among the poor . i 196
in 42 professions in Geneva i 194
in medical men . . i 200
in the liberal professions . i 196
term of, in the middle classes i 196
the conditions it depends on . v 81
the Stahlian theory of . . v 95
Life-tables, Mr. Neison's . . xxi 5
&c., by Mr. Edmonds . . iii 40
Ligament, new, of the velum palati xiii 228
Ligamenta tarsea lata . . . v 218
Ligamentous tissues, peculiarity in . iv 170
Ligaments, compendium of by Mr. M'Nab i 215
Ligamentum teres, ulceration of . xiii 169
Ligature and torsion of arteries . xxi 287
of arteries, ablation of . . xxii 96
Ligatures for arteries, on animal . xiv 24
on various . . xxii 91, 92
instrument for removing, from arteries . . . . . ii 601
of lead, in palate-fissures . xi 196
on arteries, cutting short . vi 159
subcutaneous application of . xxii 376
Light, a Manchester Chartist on . xxiii 162
bright, mode of obtaining . xix 18
Dr. Metcalfe's theory on . xviii 517
effect of, on the body . . xix 12
Light, and heat, on the sciences of . v 323
importance of, to health . v 405
influence of, on the nerves . xix 308
in relation to vegetables . vi 102, vii 176
Mr. Grove's theory on . . xxiii 248
reflected, effects of, in hydrophobia xix 301
Lightning, burning of the body by xvi 74
death from . . . . xxii 165
effects of on animals . . xviii 217
insensibility from . . . xiv 550
Ligneous principle of aliment . xvii 9, 13
Lignin, on the nature of . . iv 26
Lille, prevalence of phthisis in . xx 103
Lilliputian medical manuals . . vi 202
Limaces, internal anatomy of the . v 211
Limb, Mr. Alcock on the saving of a xi 459
shortening and lengthening of, in hip cases . . . xxii 78
Limbs, position of, in disease of joints xxi 137
Lime, carbonate of, in plants . . iv 9
chloride of, in wounds . v 575
use of, in disease . . xiii 62
hydrochlorate of, in scrofula . xxii 152
its influence on soil . . xi 441
oxalate of, urinary deposits . xvi 316
remarks on the exhibition of . viii 359
Limestone formation in relation to goitre iii 354
Limerick hospitals, report of . . v 270
Limnhemic affections, on the term . xiv 43
Lincke, Dr., on ear medicine . viii 68-9
Lincoln lunatic asylum . . ix 144, 153
Lindley, Dr., his botany . . iv 1, 2
his Flora Medica . . . vii 531
school of botany . . viii 538
vegetable kingdom . . xxii 554
Lines, French measure, remark on . viii 76
Lingual cancer . . . . xxii 294
muscles . . . . . xiv 377
Liniment of St. John Long . . iii 264
Link, M., on the Hippocratic collection xvii 463
Lion, the black, of Portugal . . x 407
Lip, lower, plastic operation on . xxii 383
Lips, cancer of . . . . xxii 293
ectropium of, operation for . xxi 304
operation for restoring . . vii 407
plastic operations on xix 404, xxi 303
Lippich, Dr., his medical notes . x 204
Lippitudo senilis urodialytica . xvii 117
Liquid aliments . . . . xvii 21
due proportion of, in food . xvii 41
food, absorbed by the stomach ii 377
Liquor amnii, analysis of . vi 248, vii 457
cases of excessive . . viii 564
chemical nature of . . vi 581
fœtus in excessive . . viii 565
nutrition from . . viii 43
placenta in excessive . viii 566
redundancy of . . iv 449
of Köchlin . . . . viii 364
potassæ, its use in phthisis . xiv 196
poisoning by . . . iv 239
puris, probable nature of . xviii 111
sanguinis, Mr. Addison on . xviii 94-6

Liquor sanguinis, or plasma . . xiv 585

Liquors, fermented, on drinking . ii 388

on the use of . . ix 243

Lisbon, and Oporto, medical schools of vi 284

hospitals in . . . . . vi 285

naval station, mortality in . xv 8

LISFRANC, arrogance and acrimony of xviii 35

fraud and subterfuge of . . xviii 32-3

charges against him . . iii 178

his clinical surgery xiv 1, xv 30, xviii 1

practice in diseased uterus i 496

remark on pathology . i 586

M. Pauly's charges against . xviii 32

on cancer, criticised . . xiv 12

on white swellings . . . ii 267

treatment of . . . ii 567

sketch of . . . . . xii 233

LISTON, Mr., discussion on a case of his xv 159-61

his case of erectile tumour . xviii 367

false aneurism . xv 155

his elements of surgery xi 305-6, 321

lectures by Dr. Mutter . xxii 379

operation for squinting . x 288

practical surgery v 457, vii 235, xxii 366

unfortunate style . . iv 147

on anasarca of scrotum . . ix 443

encysted hydrocele . . xviii 369

nævus . . . . . iii 557

plastic surgery . . . vii 386

surfaces secreting pus . xi 451

tumours of the mouth . iv 147

wounded arteries, &c. . xxiv 326

operations by, with ether . xxiii 312

Literary curiosity, a . . . ii 301

habits in medical men . . xxii 120

men, disorders of . . . iii 197

remarks on their longevity i 344

Literature, effects of, on nations . xxiv 219

medical, of the present day . xxiii 37

on style in . . . xxi 445

Lithate of soda, in relation to gout xvi 467

Lithic-acid calculus, treatment in . xi 353

deposition, remarks on . xi 352

deposits, effect of diet on xxii 224

diathesis cause of . . xxi 216

sediments in urine . . vii 162

Lithic acid, excess of, Liebig on . xv 501

in relation to gout . . xvi 465

in the blood, Dr. Jones on xvi 461

in urine, on the cause of . xi 336

Mr. Prout's theory . viii 130

over-production of . . xv 507

precipitation of . . vii 162

production of, Dr. Prout on xvi 485

solution of, in urine . viii 130

state of, in urine . . viii 131

Lithic sediments, on the causes of . xi 352

urine, mode of analysing . vii 162

yellow sediment, prevention of xi 353

Lithontriptics, contests on . . xii 394

virtues of . . . . . xii 390

Lithotome caché, disadvantages of . ii 109

Lithotome, Dr. Stevens's new . vii 506

Lithotome, M. Dupuytren's double . ii 101

Lithotomist, a Greek, in Russia, 1716 i 604

Lithotomy, after-consequences of . iv 354

and lithotrity compared . . ii 466

dangers of . . . iv 353

Mr. Key on . . . iv 350

a principal danger in . . xiv 167

at Naples, report on . . xii 254

bilateral, remarks on . . ii 110

case of, in India . . . xix 75

causes of death from . ii 107, x 372

in 19 cases . . ii 111

causes of failure of, in France . ii 106

Celsian operation of . . xvi 58

Cheselden's . . . xvi 60

danger of cutting the rectum in ii 110

dangers of, M. Velpeau on . ii 471

Dupuytren's new method of . ii 98

new operation for . . ii 101

staff for . . . ii 100

English and French compared ii 106

extent of incision in . . xiv 167

extraction of stone in . . vii 508

Frère Jacques' . . . xvi 58

hemorrhage after . . . xiv 234

history of . . . . xvi 58

the gorget in . . xvi 61

in children, caution on . . xiii 313

incision of prostate in . . x 369, 371

Indian fomentation after . xii 427

in non-calculous patients . xii 45

instruments, American . . xx 57

Dupuytren on . . ii 100

in the female, operation of . ii 259, 260

in Vienna, statistics of . . iii 254

its great success in India . xii 426

-kidney, diseased . . . xiv 166

-knife, Mr. Key's . . . ii 109

Marian operation of . . xvi 58

mode of, in Turkey . . iv 396

Mr. B. Cooper on . . . xviii 339

M. Souberbielle on . . xii 194

new instrument for . . vii 506

on healing the wound in . iv 539

penchant of surgeons for . xii 389

preferable in young boys . xiv 169

Raws' . . . . . . xvi 59

reflections on . . . xii 389

remarks of Mr. Liston on . v 468

Sir B. Brodie on . . . xiv 166

statistics of . . . ii 103-5

in Naples . . . vii 262

success of, in children . . iv 353

in Turkey . . . iv 397

urinary infiltrations in . . xxi 489

use of force in . . . vii 509

opium before . . xii 426

wounds of rectum in . . iv 353

Lithotriptic waters of Recvara . ix 246

Lithotrite, cases of failure of the . iv 351

Mr. Key's improvement in the iv 356

the screw . . . . xii 415

Lithotritic instrument, improved . v 250

Lithotrity, accidents from . . ii 469

adult age best for . . . iv 354

amongst the Arabians . . v 249

and lithotomy compared . ii 466

as an adjunct to lithotomy . iv 352

cases indicating . . . xiv 171

unfavorable for . . xiv 170

cures and deaths by . . xii 416

falsification around . . xii 395

fit state of bladder for . . iv 354

five cases of . . . . xiv 249

fragments in urethra from . xiv 171

history of . . . . xii 404

remarks on . . . xiv 168

immediate effects of . . xii 420

imminent danger from . . xii 422

in a child of 14 months . . v 250

injecting water in aid of . iv 355

instructive case of . . . iv 355

in the aged, remarks on . . iv 354

its superiority, remarks on . ii 277

medical treatment in . . iv 355

melancholy results from . xii 418

merits and demerits of . . iv 350

modes of, history of . . xiv 169

M. Velpeau on the dangers of ii 471

Messrs. Fergusson and Key on xii 419

Mr. Fergusson on . . . vii 286

Mr. Fernandez's mode of . iii 266

Mr. Liston on . . . xxii 376

an advocate for . . v 468

not valued in Italy . . i 498

number of sittings in . . xiv 171

observations on . . . xiv 172

origin of, in Italy . . . xi 270

plethoric state after . . iv 355

practical directions for . . iv 355

preparation for . . . iv 355

progress of . . . . . v 249

recumbent posture after. . xiv 172

rigors after . . . . iv 355

rival discoverers of . . xii 410

Sir B. Brodie on . . . xiv 168

tried and found wanting . xii 422

unfitted for children . . iv 353

value of, overrated . . xii 416

when admissible . . . xii 421

Lithuania, syphilis endemic in . vi 223

Lithuria, on dyspepsia as a cause of vii 161

Littell, Dr., his manual recommended v 46

on diseases of the eye . . v 31, 45

Litter for sick soldiers, new . . xxii 225

Little, Dr., on anchylosis . . xviii 353

on clubfoot . . viii 385, 405

operation for clubfoot by . vi 272

Littré, M., his cases of hernia . xiv 360

his edition of Hippocrates . xvii 450

his works of Hippocrates . xxiii 253

on artificial anus . . . xviii 459

Littré's Hippocrates, defects of . xvii 451-2

Litzmann, Dr., on puerperal fever xxi 275

Liver, abscess of, after injury of head ii 10, iii 504

disease mistaken for . ix 483

Liver, abscess of, Dr. Geddes on . xxiii 120

presenting externally . ix 276

probable cases of . . xxiii 123

puncturing . . . xii 89, 90

purulent metastasis in . xii 91

pus in the urine in . . xxiii 149

symptoms of . . . xxiii 122

(see Hepatic abscess.)

action of mercury on the . viii 361

analysis of a healthy . . xvii 437

anatomy of . . . . xxi 39

report on . . . xxi 559

and brain, sympathy between . ii 140

head sympathy between . ii 10

lungs, M. Burgraaeve on . xxiii 144

pulmonic diseases, auscultic diagnostics of . . i 487

as predisposing to disease . xx 343

cancer of . . . . xxii 300

Dr. Budd on . . . xxi 59

cells of . . . . . xviii 339

changes in, by typhoid fever . ii 50

chronic diseases of . . iii 342

enlargement of . . xiii 361

cirrhose atrophy of, described . ii 462

cirrhosis of . . . . vii 224

case of, by Dr. Huss xxii 474

complaints, in Gibraltar . . ix 65

in Malta . . . ix 65

in West Indies . . ix 64

congestion of . . . . xxi 43

Dr. Budd on . . xxi 43

on mercury in . xvii 495

consumption, bath for . . iv 254

deep-seated inflammation of . xiii 358

depuration of blood by . . xvi 214

diagnosis of diseases of . . xx 199

dimensions of, table of . . vi 140

-disease, acid bath in . . xiii 361

causing dropsy . . iii 437

from spirits, doubtful . ix 66

relative frequency of . xiii 360

diseases of, in animals . . xxiv 84

from spinal irritation i 459

in army and navy . xv 16

pain of shoulder in . vi 140

remarks on . . x 317

displaced by stays . . . i 234

distended, Dr. W. Philip on . xv 526

Dr. Budd on diseases of . . xxi 39

Dr. Thomson on diseases of . xii 78, 86

effect of hot climate on . . xii 82

effects of alcohol on . . xvi 219

enlarged, new signs of . . vii 146

enlargement of, Dr. Graves on . xvi 253

exploration of . . . vi 140

fatal hemorrhage from . . xx 243

fatty, analysis of . . . xvii 437

appearances of . . iii 155

degeneration of . xiii 553, xxii 28

disease of . . . viii 554

Dr. Addison on . . iii 155

from diseased goose-liver . xvii 25

Liver, fatty, in phthisis . . . iii 77

integumental sign of . iii 156

microscopic anatomy of . xxiv 125

particles in cells of . . xviii 339

without any tubercles . iii 155

fissure in female, from string . ii 461

function of, as a diverticulum . xxiii 143

gangrenous inflammation of . xxi 49

hydatid of, tapped . . . vii 154

hydatids in the . . . xx 411

inflammation of, resolution of . xxiii 124

inflammatory products in . . xxiv 125

interstitial deposit of pus in . iii 337

irritation of . . . . . xii 78

treatment of . . xii 84

morbid conditions of . . iii 336

M. Bonnet on diseases of . xii 78

Mr. Conwell on diseases of . iii 329

Mr. Kiernan's minute anatomy of iii 292

Mr. Mayo on steatosis of . . iii 77

pale degeneration of . . iii 343

-pathology, palmy days of . x 318

peculiar state of, in yellow fever x 500

percussion, in diseases of . . x 329

physical examination of . . xx 200

position of . . . . . xix 105

red disorganization of . . iii 343

researches in cirrhosis of . . x 560

scirrhous, state of peritoneum in iii 76

secondary abscess in . . iii 503-4

sound of hydatid cyst in . . vi 140

statistics of diseases of . . ix 64

structure of, Mr. Kiernan on . i 395

report on . . xix 274

supplementary to the lungs . xii 82

suppurative inflammation of . xxi 44

sympathetic action of brain on xii 83

tea and coffee food for the . xiv 519

tuberiform, disease so called . ii 463

tumour connected with . . xx 243

weight and dimensions of . iii 199

Livers, diseased, tropical, theory of . xx 343

enlarged, miraculous retreat of . x 329

of geese, how enlarged . . i 346

Liverpool and Manchester, mortality in . . . . . xv 304, 306

Liverpool, cause of unhealthiness of xxi 12

fever in . . . . . . xi 33

fevers from malaria in . . xviii 504

Living beings, mutations in . . xx 155

physical phenomena of . xxiii 377

by rule, examples of . . v 410

Livois, M., on the echinococcus . xvii 194

Lizards, dental development in . xx 402

Lizars, Dr., his anatomy . . ix 538

his elements of anatomy . xix 243

Lizars, Mr., flippant dogmatism of x 541

his absurdity on ophthalmia . x 538

errors on artificial pupil . x 540

jumbled, rash advice on the eye x 538

perilous eye-practice . . x 538

Practical Surgery . . vii 231

System of Surgery . . x 537

Lizars, Mr., his vague pathology of the eye x 539

incorrect language of . . x 541

incorrectness of his descriptions and figures . . . x 540

indefinite views of . . . x 541

infelicities of his style . . vii 232

quotation for his benefit . x 541

Lobster, and pagurus, Mr. Owen on the xv 212

Dr. Hall's experiments on a . v 500

Local affections in fever, table of . iv 248

Local bloodletting, Dr. Wardrop on ii 171

injuries, organic disease after . ii 14

Localities, cholera in relation to . xxiii 338

Locality for the insane, remarks on . xxii 61

Lochial discharge, appearances of . x 77

Lochia, importance of, in puerperal fever ii 93

Locke, his advice on boys' shoes . i 333

his system of philosophy . v 83

Locock, Dr. on infantile fever . xii 95

Locomotive actions, nature of . v 518

nerves of animals . . . viii 510

Lodging-houses, common, in towns xxi 345, 354

Logic, importance of a knowledge of xxiv 220

Login, Dr., on army field-carriage xxii 225

Loimology, a complete, by M. Vien i 606

Lolium temulentum in bread, danger of xviii 556

Lombard, Dr., on certain diseases . iii 510

on duration of life . . . i 194

hooping-cough . . . vii 283

effect of medicines on the heart i 264

fever in Ireland . . xii 317

nux vomica and aconite . i 249

pulmonary emphysema . vii 516

the heart . . . . iii 510

typhoid fever . . viii 428, 433

typhus fever . . . iii 260

London, advice to the sedentary in v 404

air of, Sir A. Carlisle on . . vi 187

and Manchester, mortality in xv 74

and Provincial Medical Directory xxiii 612

climate of . . . . . xii 166

decreased mortality from fever in xi 34

degeneracy of families in . vi 187

Dr. Marx's observations on . xv 21

extent of pauperism in . . xiv 455

fevers of . . . . . xi 5

in, from malaria . . xviii 503

getting on in, Dr. Marx on . xv 27

improvement in the health of xi 35

medical men of, Dr. Marx on . xv 22, 29

mortality of children in . . vi 187

new bills of mortality for . ix 582

prevalence of fever in . . xi 32

remark on the temperature of xiv 472

reports on malaria in . . xi 5

table of mortality for x 589, xi 35, 267, 545, xii 285, 575, xiii 267, 577

London College of Surgeons iii 292, xii 576

London Hospital, statistics of . iii 270

London Medical Dictionary, Dr. Parr's i 177

London Pharmacopœia iv 101, xiii 54

London University, charter of . iii 293

examiners of . viii 304

London water, qualities of . . xviii 496
Long- or far-sightedness, lenses for xvi 362
LONG, ST. JOHN, purchase of his secret xxii 89
LONGET, M., on inhalation of ether xxiii 570
on muscular irritability . . xiii 226
on roots of nerves . . . xii 239
Longevity and mortality in Prussia viii 575
and regimen, Dr. Bell on . xviii 115
connected with happiness . i 319
constitutional signs of . . xvii 105
effect of active life on . . i 198
civilization on . . xvii 104
sedentary life on . . i 198
in families, Mr. Travers on . ii 6
influenced by habits . . i 198
influence of riches on . . i 195
in Russia, statistics of . . x 582
in town and country ix 357-59, xviii 199
of classes of men . . . xvii 104
of literary men, remarks on . i 344
of medical men, Dr. Caspar on i 200
of printers . . . . . i 284
what most conducive to . . iv 62
why affluence favorable to . i 196
Longings in pregnant women . viii 17
LONSDALE, Dr., on hydrocyanic acid vii 572
LONSDALE, Mr., on curvature of the spine . . . . . xxiv 578
on fractures . . . . . vi 308
Lophius (frog-fish), nervous system of v 495
LORD, Mr., his physiology . vii 214, 216
Lordosis, how produced . . v 137
LORIMER, Dr., his report on cholera xxiii 313
LORINSER, Dr., on the plague . v 191
Lotion, sedative, for headache . xi 235
cold, improved mode of applying ii 199
Lotions, Dr. Granville's . . vii 292
ophthalmic . . . . . xi 57
LOUDON, Dr., on population . . xvi 229
LOUIS, M., Gavarret's censure of . xii 18
his censure of past observers . v 155
declamatory language . v 160
influence on his pupils . vi 29
mode of investigation . v 156
mode of studying diseases . i 168
researches on phthisis . i 165
work on gastro-enterite . vi 41
illustrious name . . xvi 460
numerical method i 170, 404, iii 459, v 155
on bloodletting . i 397, 405, iii 456
clinical instruction . . i 397, 400
consumption . . . . xvi 425
emphysema of the lungs . vi 28
fever, misrepresented . xii 33
phthisis, new translation of xviii 526
professional history of . i 168
typhoid fever . . v 549, xii 22
typhus fever . . . xii 294, 318
yellow fever . . . . x 494
zeal and merits of . . xii 53
Louisiana, (U. S.), Medical College of ii 591
LOUVRIER, his plan with contracted knee . . . . . xiii 14, 16
LOUVRIER, his treatment of anchylosis xii 552
Love, its connexion with hysteria . iii 401
observations on the nature of . vii 374
physical, effects of on nervous system xii 64
remark of a German author on vii 374
ridiculous, Italian case of . i 547
symptoms of, described . . v 404
LOWDELL, Mr., on hydatid cyst . xxiv 328
Low diet, bad effects of protracted v 389
LÖWENHARDT, Dr., on ovarian inflammation . . . ii 526
on practical medicine . . viii 419
Lowering the system, power of hydropathy in . . . xxii 446
Lower jaw, excision of . . . vii 152
Loxarthus, or clubfoot, lecture on xi 513
LUCAS, Mr., on strabismus . . xi 474, 492
Lucid interval, Dr. Conolly's test of a x 135
intervals, conviction of crime in xvi 86
mesmerism, remarks on . . xix 449
Lues uncaused by contagion, remarks on i 372
venerea, (see Syphilis.)
LUGOL, M., on scrofula . . . xix 239
on scrofula, strictures on xviii 491-92
on the causes of scrofula . xviii 481
LUJEFF, Dr., and the Prince of Russia, 1534 . . . i 598
LUKE, Mr., on strangulated hernia xviii 367
Lumbago and pyelitis, diagnosis of xv 474
case of apparent . . . vi 357
M. Piorry's theory of . . vi 146
Lumbar muscles, case of discoloured vi 357
Lumbrici, frequency of in China . xxii 186
remarkable case of . . . vii 224
Lumbricus teres, separate sexes of xviii 320
Lunacy, on commissions of . . x 163
law of, a barrister's letter on x 129, 163
change required in . . xxii 62
letter on the law of . . xvi 81
present state of law of . . x 129
report of commissioners on xix 317, 343
Lunar caustic in follicular disease xxiv 497
in gonorrhœa in women iv 258, 259
influence, Dr. Laycock on xviii 171
on delirium, remarks on . xx 22
on disease . . . viii 481
Lunatic asylum, a bad one, at Geneva i 498
at Berlin, bad . . i 500
at Florence, good . . i 498
at Naples, statistics of . vi 238
at Rome, bad . . . i 498
at Sonnenstein, good . i 499
chaplain in a . . . xii 56
construction of . . ix 180
County of Middlesex . ix 156
Esquirol's plan for a . ix 150
improved plan of . . i 308
in Pennsylvania . . viii 583
Morningside, music in . xv 282
picture of a proper . . v 74
printing, &c., in a . . xviii 509
Lunatic asylums, admitting pupils to vii 34, 43
airing-grounds in . . ix 182
as they were and are . v 70-1

Lunatic asylums, attendants in xxii 358, xxiv 163

beds or couches in . . ix 169

bedsteads in . . . xi 122

cells for the furious in . ix 153

classification in . . ix 179

construction of . . xi 122

county, pauper . . xix 319

cruel economy in . . xxiv 159

diet in . . . . ix 167

dysentery in . . . vii 49

Dr. Conolly on . . xxiv 156

economy, &c., of . . xxii 357

employments in . . ix 173, 182

former state of . . ix 146, 164

division in . . . ix 179

dormitories in . . xix 284

evils of restraint in . . ix 156

general remarks on . . ix 145

government of vii 48, ix 183, xix 600

housekeeping in . . vii 50

in France . . . ix 152

inspectors of . . . vii 55

in Turkey . . . iv 390

keepers of . . ix 177, 180

land to . . . . xix 286

management of . . vii 30, 42

matrons of . . . ix 184

minute attentions in . xxiv 158

mortality in . . xxii 350, 360

Mr. Browne on . . v 65

neglect in, effect of . vii 42

observations on . . xxi 464

officers of . . . ix 176

on heating of ix 170, 181, xix 286, 320

on scenery around . . xxiv 157

pauper, mortality in . xxii 353

physicians of . . . ix 176

plays and dances in . ix 175

privies in . . . ix 169

pupils in . . . xiv 218

reception-room of a . ix 179

recoveries in . . . xxii 360

religious services in xii 58, xxiv 164

reports of . . . vii 1, 2

reports from . . . vii 27

sites of . . . . vii 41

Sunday in . . . ix 185

superintendents of vii 55, ix 188, xxiv 164

shocking details of . . v 70

value of labour in . . ix 172

various authors on . . ix 144

various kinds of . . xix 319-23

windows in . . . ix 169

work done in . . ix 171-74, 186

epileptics, on securing . . xi 122

gentleman, narrative of a . xi 104

hospitals, management of . vii 30, 42

Lunatic, account of his insanity by a xi 107

case of an American homicidal ix 164

in Ceylon, case of a . . xix 608

late extension of the term . x 131

treatment of one at Hanwell . ix 166

Lunatics, admission of, into asylums xix 327, 340

agricultural labour by . . ix 175

amusements for . . . ix 175

arguments for severity to . xi 123

association with each other . xi 115

at Haslar, boating of . . xvii 285

fishing by . . . xvii 287

attention to dress of . . xxiv 162

benefit of asylums to . . xi 124

classification of . . . xix 332

coercion-chairs for . . . ix 159

convalescents as keepers to . ix 177

criminal . . . . . xix 341

cruel restraint of . . ix 147, 164

curable and incurable . . xix 329

depravity of . . . . . xii 54, 60

destruction of clothes by . ix 159

diet of . . . . . ix 167

diseases of . . . . vii 30

douche bath for . . . ix 178

Dr. Conolly on treatment of . xiii 274

Dr. Winslow on the Act for . xxi 276

dress for destructive . . ix 159

dress of . . . . . ix 168

effects of idleness on . . xi 113

epileptic, treatment of . . ix 157

farm labour of . . xix 295, 333

feeding of . . . . ix 168

female, employments of . . xxi 461

former bad treatment of ix 146-49, 164, 185

severity to . . . xix 296

formerly in Lincoln Asylum . ix 164

homicidal, Mr. Taylor on . xxi 486

imbecile and helpless . . xix 288

importance of the charge of . ix 188

injudicious language to . . xi 111

in Norway, official report of . i 278

in Pennsylvania, statistics of . viii 584

in the United States, state of . xv 55

knives and forks to . . ix 168

labour for . . . . ix 170

law on confinement of . . x 174

mean age of . . . . vii 22

means of opening the mouth of ix 168

medical treatment of . . xix 330

mild means of restraining . ix 178

mode of restraining violent . vii 44

motives to crime in . . xvi 90

music to . . . . . ix 175

naval, at Haslar, treatment of xvii 285

occupation of . . xix 295, 333

on trusting tools to . . ix 175

paralytic, remarks on . . vii 41

pauper, on profit from . . xix 325

expense of . . . xix 319

Pinel on moral qualities of . xii 60

progress of non-restraint of . xi 116

propensity to suicide in . . xi 120

public management of . . vii 55

recreation of . . . . xxi 463

refractory or noisy . . . ix 170

refuge for discharged . . xix 292

refusal of food by . . . ix 167

Lunatics, religious instruction to . xii 54
services to . . . . xix 339
responsibility of, Lord Erskine on x 141
respect to be shown to . . xi 155
restraint of . . . . . xix 334-39
abolition of . . ix 154, 167
on pleas for . . xix 607
tabular view of . ix 154
remarks on . xix 283, 297
or non-restraint to . xi 104
sketch of inappetent . . vii 10
space and exercise to furious . ix 169
state of, in Wales . . . xix 342
table of mortality of . . vii 22
temporary restraint of . . ix 155, 158
thickness of skull in . . v 373
total disuse of restraint of . xv 218
treatment of . . . . . xi 104
unchaining of, by Pinel . . i 286
uncleanly, remarks on . . xix 288
violent, treatment of . . xi 546
vomiting mostly difficult in . ii 542
Lung, atrophy of, by compressed bronchia ii 465
carnification of . . . xviii 338
concentric force of . . vii 354
different sounds of right and left iii 188
cyanosis from absence of right vi 220
excavated, pectoriloquy a sign of iv 322
excavation of, the seton in . iv 324
treatment of . iv 324
projecting from wound, cured. iv 153
rupture of, from severe cough . iv 254
signs of ulceration of . . iv 322
solidification of, without signs iv 315
solidified, case of . . . ix 490
clear sound in . . ix 490
mucous rattle in . . ix 490
weight of healthy and hepatized xiii 383
Lungs, abscess of . . . . . xx 246
pointing distantly. xiv 579
remarks on . . xiii 557
active congestion of . . ix 321
affected by sexual debauchery ix 324
air-cells of, Mr. Addison on . xvii 94
air in after death . . . xvi 149
anatomy of black . . . iv 150
Dr. Addison on . . xiv 59
and air-tubes, contractility of . xi 218
and heart, relative position of i 428
and liver, M. Burggraeve on . xxiii 144
and thorax, changes of, in the aged i 233
artificial dilatation of, test of . vi 63
inflation of . . . xvi 310
atrophy of, from emphysema . ii 461
black matter of . . . i 135
buoyancy of, Mr. Jennings on ii 413
cancer of, different forms of . xv 430
Dr. Stokes on . . xiv 242
Dr. Walshe on . . xxii 300
frequency of . . . xv 430
physical signs of xv 434-38, xxii 302
symptoms of . . . xv 433
ulceration of . . xv 432, 438
Lungs, cancer of, works on . . xv 430
capacity of . . . . . xxiv 327
for breathing . . xvii 256
carcinoma of, case of . . xx 90, 253
carnification of . . . . xv 555
in children . . . xii 355
in infants . . . xxiii 303
caverns or excavations in . xxiii 431
cavities in, distinction of . xvi 427
M. Louis on . . xvi 427
cells of, their size in old age . i 235
changes of, in typhoid fever . ii 50
circulation in, report on . xix 263
cirrhosis of . . . . . xx 227
compression of, use of as a test vi 63
condensed, cellular tissue in . xvi 428
congestion of, diagnosis in . xv 88
crises effected by exhalation from i 246
defective expansion of . . xx 213
density of, diminishes with age i 235
depression at upper part of . xvi 429
diseases of, delirium in . . xx 19
diagnosis of . . . xv 223
from dust, &c. . . xviii 302
from spinal irritation . i 459
in army and navy xv 11, xvii 317, 320
in Barbadoes . . . viii 219
in Mediterranean . . viii 220
in North America . . xvi 42
in pregnancy . . . xxi 90
in West Indies . . viii 219
in the Guards. . . viii 225
relative prevalence of . vi 21
displacement of by stays . i 234
Dr. Stokes on diseases of . iv 285
effect on, by injection of mercury viii 323
effusion in, error of Dr. Hall on xiv 56
elasticity of, theory on . . xi 449
emphysema in, causes of i 482, xv 87
of, on danger in . . xviii 163
effects of on chest . vii 364
M. Prus on . . xviii 163
pathognomonic sign of vii 365
remarks on . . i 22, 6
site of air in . . xviii 163
sudden death in. . xviii 163
emphysematous, in infanticide iv 91
engorgement of . . xi 406, xiii 383
fibrous bands in, in phthisis . xxiii 435
fœtal, average weight of. . v 180
formation of air-cells of the . xiv 573
gangrene of the . . xi 408, xx 226
cases of . . ii 547, 548, xx 247
in the insane . . . ii 546
not incurable. . . . ii 548
gray granulations in the. . xvi 426
nature of . . iii 64
hepatization of . . xi 407
hemorrhagic hepatization of . iii 259
hepatization of, exudation in . xxiv 123
in children . . . xxi 392
hyperemia of the . . . ix 326
indurated, percussion at fault in ix 490

Lungs, inflammation of, in rheumatism xx 184

inflammatory products in . xxiv 123

inflated, death from . . vii 519

in relation to infanticide . xvi 68

insufflation of, in infanticide . ii 413

lobular passages of the . . xvii 94

malignant disease of, cases of . xiv 131

minute structure of xiv 546, xxiii 415

mode of ventilation of the . xvii 153

inflating . . . xxii 163

morbid changes in from nerves viii 266

M. Piorry on exploration of . vi 137

obstructed circulation in . xxi 266

œdema of, respiration in . vii 263

of infants, experiments on . vi 62

of newborn infants, weight of ii 410

of the old, congestion of the . ii 544

granulations in . . ii 545

gray hepatization of . ii 544

red hepatization of . . ii 544

texture of . . . iv 311

opiate liniments in diseases of xv 410

point of contact of the . . xix 104

position of the . . . xix 104

predisposing causes of disease in xx 344

red hepatization of . . xi 406

relation of the liver to the . xii 82

relative affection of the two . ix 334

renewal of circulation in . xxi 268

resident air in, volume of . xvii 153

sanitary effects of sea life on . xv 15

secondary abscess in the . iii 503-4

cancer of the . . . xv 431

severe wound of, cured . . xiv 255

signs of excavation of . . ix 311

size of their cells at different ages i 235

static tests of . . . xiii 572

structure of the . . . xxi 552

report on . . . xvii 256

supplementary air of, volume of xvii 153

suppuration of, in the old . ii 544

their importance in nutrition . i 246

their use in assimilation . i 246

three diseases of, from scrofula xvi 252

tubercle of, with emphysema . xi 410

tubercles in, Dr. Hodgkin on . xi 409

tuberculous, on new vessels in xvi 429

on purgatives in . . xiii 367

products in . . . xxiv 127

ulcer of, cured by creasote .. ii 168

very forcible inflation of, fatal. vi 63

watery vapour from . . i 241

weight of, in newborn infants xvi 309

of uninspired infant . vi 63

wound of, emphysema from . xxii 324

Lupus, nature and treatment of . iii 556

on the treatment of . . xv 36

use of creasote in . . . vi 270

iodine in . . . viii 522

Luscitas of the eye . . . xi 482

Lusus naturæ, remarkable . . vi 381

Luxated humerus, new mode of reducing iii 241

a mode of reducing . vi 163

Luxation, congenital, functional . xxi 399

of humerus . . . x 566

of lower jaw . . . xiv 238

of the ribs . . . xxi 401

of elbow, reduction of old . vi 233

of humerus, compound, case of iv 256

dissection of a . . xii 455

on incomplete . . xii 455

obstetrical, diagnosis of . . xxi 396

original . . . . xxi 402

diagnosis of . . . xxi 406

hereditary. . . . xxi 407

remedies for . . . xxi 407

spontaneous, congenital . . xxi 396

of vertebra, reduction of . x 567

Luxations, congenital causes of . xxi 394

obstetrical . . . xxi 39

treatise on . . . xxi 393

of femur, on the pelvis in . iii 240

spontaneous . . . xiv 554

cure of . . iii 129

Luxford, Mr., his phytologist . xxiv 436

Lying-in Charity, Guy's . . xii 343

Hospital, at Copenhagen . iii 568

at Prague . . . xiii 109

what it ought to be . vii 494

hospitals, construction of . xiii 103

women, causes of death in 164 ii 74

deaths among 16,434 . ii 73

on bandaging abdomen in iii 267

treatment of . . . iv 399

Lymbisanus, Horatius, on plague. xiii 497

Lymph, and its corpuscles . . xv 272

coagulable, composed of oxyprotein xviii 263

coagulated, in primæ viæ in cholera i 238

constituents of . . . vi 546

-corpuscles, function of . . xviii 78

structure of . . . xiv 262

-globules and vesicles, formation of xvi 225

of birds . . . . xiv 571

on motions of the . . vi 219

nucleated cells . . ix 509

human, analysis of. . . vi 544

M. L'Heritier on . . . xvii 427

microscopic examination of . xii 528

morbid alterations of the . iv 347

motion of its globules . . iv 500

nature and composition of . iv 337

of blood, coagulability of . viii 273

pathology of interesting . viii 273

plastic, effusion of . . . vii 434

primary, vaccination of cow with x 468

properties of . . . . xix 278

propulsion of . . . xxi 565

rapid organization of . . xi 458

vaccine, kind of, to be used . xiv 402

on new and old . . xiv 402

Lymphadenitis, idiopathic, case of . xi 241

Lymphangioitis, uterine . . xiii 114

Lymphatic and lacteal absorption . xix 278

glands, inflamed, causes of . ii 559

inflammation of . . ii 558

remarks on . . . iv 333

Lymphatic glands, resolution of inflamed ii 560

structure of . . iv 334, xx 297

suppuration of . . ii 560

treatment of inflamed . ii 560

tuberculization of . . xvi 434

uses of the . . . iv 343

habits, the lymphatics in . vi 135

hearts, report on . . . xix 279

Volkmann on . . xxi 565

system, the, on diseases of . ii 556

diseases of in animals . xxiv 84

inflammation of . . xiii 174

M. Breschet on the . iv 325

physiology of . . . iv 340

predisposing to disease . xx 341

temperament . . . . xv 425

Dr. Lévy on . . . xix 54

Lymphatics, absorption by, questioned viii 312

absorption of gases by . iv 342

and lymphatic ganglia . . xxiii 142

and veins, communication of . iv 335

case of dilatation of the . . ii 459

case of disease of . . . iv 348

comparative anatomy of . . iv 326, 339

contraction of the . . . iv 346

dilatation of the . . . iv 344-5

case of . . . . . iv 345

elective absorption by . . xvii 259

fungous growths in . . iv 347

general structure of . . xiv 289

hypothesis on the . . . iv 326

in fishes, M. Fohmann on . iv 336, 339

inflammation of the . ii 556, iv 346

in birds . . . . . . iv 340

placenta and cord . . iv 328, 338

reptiles . . . . . iv 339

brain, detected . . . iv 327

mammifera . . . . iv 340

literary history of the . . iv 325

medicines influencing . . viii 356

melanotic matter in . . iv 347

minute anatomy of . . iv 329-31

ramifications of . . iv 329

network of . . . . . iv 329, 331

of the intestines . . . iv 337

of tadpoles on globules in . iv 500

on mistaking veins for . . iv 327

ossific deposits in . . . iv 347

presence of blood in . . iv 341

production of disease by . iv 346

propelling force in . . . iv 343

pus in the . . . . . iv 346

source of their origin . . iv 328, 330

structure of . . . iv 332, xxi 564

tubercular matter in . . iv 346

the valves of . . . . iv 333

variable size of the . . iv 332

Lytton, Sir E. B., his Confessions of a Water-patient . . . xxii 428

M.D., after a name, on the title to . xxii 549
M'Arthur, Dr., his directions to seamen xxiv 212
Macartney, Dr., on inflammation . vii 221, 417, 426, viii 169, 184
on sounds and actions of the heart ii 598
on structure of the brain . . ii 129
strictures on his style, &c. . viii 185
Macaulay, Mr., on cruelty to animals viii 536
M'Clelland, Mr., his zealous researches viii 105
on bronchocele . . . viii 103
M'Cormac, Dr., his Methodus Medendi xv 534
on continued fever . . . i 172
M'Divit, Dr., obituary of . . ix 296
Macduff, on the birth of . . xii 489
Maceration of organs, effects of . viii 124
Macgregor, Sir J., on the expedition to Egypt . . . vi 153
Machines, animal or biotic . . xxiv 183
bodily, operations of the cerebral powers on . . . xxiv 194
of the body, Unzer on . . xxiv 194
Macilwain, Mr., his law of inflammation vi 112
obscure passage from . . vi 100
on errors in diet . . . ii 182
his own success . . . vi 102
medicine, &c. . . . vi 98
tumours . . . . . xx 127
unity of the body . . ii 180
remarks on his style . ii 183, vi 99
samples of his reasoning . . vi 101, 111
Mackenzie, Dr., on diseases of the eye ix 543, x 3, xx 517
on fever in Glasgow . . xviii 179
glaucoma . . . . vi 274
Nature in diseases . . xxiii 585
strabismus . . . . xii 520
syphilitic ophthalmia . . x 381
vision . . . . . xii 217
Mackinnon, his bill on health of towns . . . . . xv 513, 523
Mackintosh, Dr. John, obituary of v 311
his axiom in physic . . iii 103
his practice of physic . . iii 101
his work, American edition of . iii 102
Mackintosh, Sir James, saying of . v 201
Macleay, Mr., ingenious principle of iv 9
his system of animated nature . xix 181
Macleod, Dr., his report from St. George's iii 552
on rheumatism . . . xiii 445
Maclise, Mr., his plates of the arteries xi 211
Maclure, Mr., his Collectanea Medica xxii 226
M'Nab, Mr., his compendium of the ligaments . . . . i 215
Macnaughten, case of, Dr. Mayo on xix 38
defence of, remarks on . . xvi 101
his trial for murder . . xvi 81, 101, 274
Mackness, Dr., his Moral Aspects . xxiii 467
his translation of Marx . . xxiii 470
Macnish, Dr., death of . . iii 586
Macreight, Dr., his British Botany vi 511
on mesmerism . . . . vii 349
Mac Robin, Dr., his Practical Medicine i 203, 207
Macula lutea of the eye, on the . x 254
Maculloch, Dr., his saying of Italy i 351
on malaria . . . i 349

M'William, Dr., on the Niger expedition xvi 259

Madagascar, on the tribes in . . xxiv 469

Madar plant, its use in diseases . xix 74

Madden, Dr., on cutaneous absorption vi 332

Madder-experiments on bone . . xxiv 406

Madder, its action on the bones . x 257

its influence on bones . . ix 272

Maddock, Dr., on copaibal rheumatism viii 280

Mad dogs do not always dread water v 262

Madecassian, or Malecassian race . xxiv 469

Madeira, on the climate of . . xii 171

statistics of phthisis in . . xxiv 107

Madhouses, Dr. Crowther on vii 2, 48, xi 515

Madman, fettered, rupture of spine by xiii 542

Madness, acute, rare in Turkey . iv 390

a poem on . . . . . xiii 412

Dr. Ferrarese on . . . vii 1, 50

in animals (see Rabies.)

in families, misery from . . ix 187

in the honest labourer . . ix 187

in the rich and poor . . ix 187

predisposing causes of . . ix 187

reflections on . . . . ix 186

Madras European regiment, diseases in xxiii 109-28

Madras Medical Journal . xiii 347, 364

Quarterly Medical Journal . xii 78, 89

Magazine, New, of Zoology and Botany ii 301

Rust's Medical . . . i 545

Magdeburg, medical school of . i 299

Magendie, his Compendium of Physiology . . . . . xiii 467

and his vivisections . . viii 149

general remarks on . . viii 351

his complaints of neglect . . xiv 462

discoveries on the nerves . ix 132

experiments, strictures on . ix 291

formulary of new remedies . ii 207

hydrophobic experiment . xiii 53

mutable inferences . . viii 310

irony and conceit of . . viii 338

on cerebro-spinal fluid . . xiv 463

endosmosis of the egg . ix 290

life . . . . . viii 309, 352

physical science . . v 319

spinal nerves . . . ix 132

strictures on . . . . viii 537

on his reasoning . . viii 309

Magistral blister of M. Valleix . xviii 289

Magma reticula in the ovum . . xvi 556

Magnesia, effervescent . . . vii 570

Magnesium, as an element of food . xvii 4

Magnet, the, a cure for gout . . ii 246

effects of its application . . viii 481

surgical use of the . . . xiv 557

Magnetic, animal, emanation . . vii 324

fluid, doctrine of a . . . vii 325

model for church service . . vii 338-9

pharmacology . . . vii 317

sleep . . . . . . xix 432

remarkable case of . . v 599

somnambulism . . . vii 331

state, explanation of the . . viii 494

Magnetic traction, observations on . xix 480

Magnetism, and electricity, cures by xvi 269

animal . . viii 494

Dr. Sigmond on . . v 598

history of . . . vii 303

Laplace on . . . vi 196

Mr. Colquhoun on . . iv 441

remark on . . . iii 328

treatises on . . . vii 301

Magnetising an apricot tree . . vii 321

from a separate room . . xix 478

of women described . . vii 309

Magneto-electric apparatus, Keir's . xvi 269

machine of Mr. Hearder xxiii 406

-electricism, Dr. Wetzler on . xvi 269

-electricity in ear-diseases . xxiv 40

Magniloquence, American medical . iv 194

Magrath, Mr., on oxide of bismuth iii 217

Magyars, or Hungarians, descent of . xxiv 461

race of, on the skulls of . . xxiv 75

Mahon, M. M., secret depilatory of ii 153

Maillot, Dr., on intermittent fever viii 56

Maine (U. S.), medical school of . ii 589

Maison d'Accouchement, account of i 281

midwifery cases in the i 588

d'Aliénés . . . . . ix 151

Royale de Santé, account of . i 281

Maisonneuve, Dr., on coxalgia . xxi 124

Maize, nutritious elements in . . xvii 19

Majendie on pulmonary vapour . i 241

on watery vapour from the lungs i 241

Make-shift surgery . . . iv 232

Maladie charbonneuse . . . xiii 41

Malapert, M., his artery-compressor xvi 64

his new treatment of bubo . x 418

Malapraxis, jurisprudence in . . xvi 72

Malaria, and marsh fever, remarks on xiii 350

cases showing the effects of . iv 162-3

does not produce fever . . xi 22, 36

doubts of the existence of . xviii 222

effect of, on infant life . . xviii 511

effects of, on lower animals . iv 163

from salt marshes . . . xiii 350

generation of . . . iv 161

importance of the subject . iv 161

in relation to climate . . xiii 257

to drunkenness . . xviii 505

to infanticide . . . xviii 504

to morals . . . xviii 504

in Liverpool, fevers from . xviii 504

in London, Dr. Arnott on effects of xi 8

many ailments from . xi 8

reports on . . . xi 5

its active principle . . xx 209

marsh, on the nature of . . xiii 351

modification of diseases by . iv 162-3

neglect of study of, in England iv 161

on fever in Glasgow from xviii 505, 509

on fevers in London from . xviii 503

resisted by woollen clothing . i 332

various authors on . . i 349

Malarious atmosphere, diarrhea from xviii 510

localities, diet in . . . iv 162

Mal-assimilation, on diseases from . xi 336
Malayo-Polynesian nations . . xxiv 467
Malays, proper, description of . xxiv 467
MALCOMSON, Mr. on Beriberi . v 116
on enlarged liver . . . vii 146
liver abscess . . . ix 276
solitary confinement . . iv 491
MALDEN, Dr., his address . . ix 216
his speech at Cheltenham . iv 559
Male animals, pugnacity of . . xii 64
MALE, Dr., his death from aconite xx 469
his work on forensic medicine xx 471
life and character of . . xx 470
Males and females in England, deaths of xi 424
MALESPINE, on absorption of sequestra xv 30
Malformation, of urinary organs . xx 511
original . . . . xxi 402
singular case of . . . viii 566
Malformations, causes of congenital viii 16
congenital, groups of . . viii 18
MALGAIGNE, M., his lectures on hernia xiv 91, 110
his Operative Surgery . . xxiii 155
on capital operations . . xv 240
dislocations . . i 583-5, vi 231
fractured ribs . . . vii 554
hernia . . . . . viii 561
surgical anatomy . . x 347
MALIBRAN, Dr. Wetzler on the death of vi 515
Malignancy of tumours, remarks on v 415
Malignant, Mr. Hawkins on the term ii 162
diseases, definition of . . vii 143
of head and face . . xiv 580
pustule on eyelids . . . . x 26
tumours, diagnosis of . . xv 191
Malignity of growths, Dr. Carpenter on xiii 165
Maligners, difficulty of managing . xviii 119
surgeons' conduct to . . xviii 122-3
Malingering, motives to, in soldiers xviii 118-9
trick with urine . . . viii 132
MALLE, Prof., his Clinical Surgery x 49
Malleolus, external, excision of . xv 400
MALLET, Mr., on hernia cerebri . ix 220
Malleus of ear, position of . . viii 75
Malpighian bodies, in the spleen . xxiii 142
structure and use of xiv 567
capsules, report on . . xxii 267
MALPIGHI, his discoveries . . xiv 482
his glands in Bright's disease . xiii 436
Malposition of kidneys, case of . xiv 61
Malta, bowel complaints in . . ix 67
climate of . . . . . xii 171
diseases of lungs in . . viii 221
history of the plague at . xvi 292, 295
prevalence of phthisis in . xiii 434
MALTHUS and his theory, remarks on xxii 341, 348
Malthusian theory, coup de grâce to the xvii 406
Malum regimen, case illustrating . v 177
Mamelukes of Paraguay . . . x 447
Mamma, hypertrophy of the . . iv 224
inflamed, emetic tartar in . iv 533
Mammæ, absorbents of . . . x 119
and genitals, sympathy between x 111
Mammæ, and nipples, females with four v 585, 594
uterus, sympathy of . xxiii 192
arteries and veins of the . x 118
atrophy of, on menses ceasing ii 465
changes in from age . . x 110
diseases of . . . . . xxii 156
cupping-glasses to, in uterine hemorrhage . . . xxii 156
engorgement of . . . vi 185
evolution of . . . . . x 122
excretory ducts of . . . x 115
hypertrophy of . . . xxii 156
influence of gestation on . x 123
in the inguinal region . . viii 24
mucous secretion of . . x 109
nerves of . . . . . v 119
on suckling by male . . x 111
supernumerary . . . viii 24
varying consistence of . . x 109
Mammalia, cerebellum in . . xxii 540
development of, *plates of* . xviii 233
domestic, physiology of . . vi 513
natural history of . . . xxi 496
Mammalian palæontology, remarks on xxi 478
Mammals, number of teeth of . xxi 63
odontography of . . . xxi 62
Mammary abscess, compression in . xii 259
areola, venous circle of . . vii 240
fascia, description of . . xx 135
gland, anatomy of . . . x 116
comparative anatomy of . x 128
male, function of . . x 122
structure of . . x 121
milk-cells of . . x 116
of the male, use of . . x 110
glands, position of the . . x 106, 108
or lactiferous tubes . . x 116
Mammiferous animals, diffusion of iii 369
Mammillary, nervous apparatus of skin ii 432
Man, and animals, diseases of, compared xxiv 82
animals, and plants . . xiii 473
an omnivorous animal . . xvii 27
farinacea and fruits the food of xxi 148
Hippocrates on the study of . vi 298
his descent from one stock . iii 371
his true relation to nature . xii 9
Mongolian type, the original of xxiv 479
M. Quetelet's essay on . . iii 40
natural history of xiii 521, xv 180, xxiv 49
origin of, observations on . xix 176
physical history of . . iii 365
report on the . . xvii 274
physical necessities of . . xiv 447
physiological anatomy of . xx 201
popular lectures on . . xiii 222
psychical position of, in creation xix 311
rejuvenescence of . . xvi 208
the fountains of happiness in . i 321
uninstructed, his ideas of God iv 56
varieties of, from climate . xv 181
physical likeness of . iii 373
Manchester, and Liverpool, mortality in xv 304, 306
and Rutlandshire, deaths in . xv 305

Manchester, decrease of fever in . xi 32, 34
its factory population . . xiv 458
Medical School . . . i 206
Mr. Noble on phthisis in . xv 310
Mandibulitis adultorum . . viii 424
MANDL, Dr., his Microscopic Anatomy xiv 478, 480
on analysis of the blood . . xii 270
on the blood . . . xi 225
on the microscope . . xiv 478, 480
Manganese, in the hair, bile, &c. . xvii 5
poisonous effects of . . iii 552
Mange, or scabies ferina . . xiii 49
transmission of, to man . . xiii 49
Mania, acute, or maniacal furor . xx 19
after injuries of the head . xv 168
and delirium, diagnosis of . x 132
and encephalitis, connexion of xx 20
a regular course in . . vii 16
bleeding in . . xi 543, xiii 559
cause of its rarity in Turkey . iv 390
cold douche in . . . xx 37
compression of carotids in . xxii 14
diversities of conduct in . vii 15
fatal termination of . . xx 20
feigned diagnosis of . . x 136
furious, in epileptics . . x 171
homicidal, from impulse . . xvi 109
jurisprudence of . x 138-47, 167
two forms of . . xx 27
irregular progress of . . vii 16
morbid anatomy of . . xx 21
more curable than other forms vii 18, 21
more frequent in men . . vii 24
of epileptics, peculiar . . vii 17
of temper, description of . xx 28
on impaired attention in . vii 18
period of crisis in . . vii 16
persons most subject to . . vii 16
preceded by melancholy . . vii 17
precursory symptoms of . . vii 16, 17
reasoning described . . xx 28
second period of . . . vii 16
seldom sudden . . . vii 17
sensorial functions in . . vii 17
shedding tears in . . . vii 31
state of mental faculties in . vii 17
sudden case of . . . x 134
symptoms and character of . vii 15
the physical functions in . vii 18
tolerance of cold and hunger in vii 15
with delirium, case of . . xxii 5
with illusion, cases of . . xxii 7, 9
without illusion, case of . . xxii 3
Maniacal attacks, on pyrexia in . xxii 13
Maniacs, Pinel on moral qualities of xii 60
Mankind, authors on the diversity of iii 366
changes in, on time in relation to xxiv 480
colour of the skin of . . xxiv 68
commonness of diseases to . iii 373
Dr. Prichard on . . . v 543
Dr. Prichard's classification of iii 374
ethnographical inquiry on . iii 366
improved by mixture of races . iii 373
Mankind, moral identity of . . iii 374
original seat of . . . xxiv 479
physical history of . . xii 519, xxiv 441
picture of the variety of . . iii 366
primary source of . . . xxiv 479
psychical characteristics of . iii 374
phenomena in . . xxii 16
races of, observations on . xviii 387
physical identity of . xxiv 478
unity of the species of . iii 365, v 543
varieties in skeletons of . . xxiv 71
varieties of . . . . iii 375
MANNI, Prof., on periodical maladies viii 57
Mannite, account of . . . ii 208
MANSA, Dr., his Medical Journal, Danish ii 231
Manual of Anatomy, by Dr. Krause iii 198
Manuals, for students, remarks on . i 512
Mr. Churchill's . . . xxi 479
on Lilliputian medical . . vi 202
Manufactories, diseases in . . xv 300
influence of, on health . xv 286, 300, 313
noxious vapours from . . xix 4
woollen, on health in . . xv 287, 298
Manufacture of noses . . . xxi 294
silk, on health in . xv 299
Manufactures, influence of, on health xvi 507
the philosophy of . . . xv 285
Manufacturing districts, scrofula in xv 312
poor, habits of the . . . viii 524
towns, improvement of . . xiv 458
in France . . . xiv 456
operatives in . . . xiv 459
Manures and soils, remarks on . iv 16
Dr. Lindley on the action of . iv 16
Liebig on the value of . . xvii 225
necessity of phosphates in . xvii 226
Manuring with sewer-water, profit of xxi 358
Manuscript, oldest medical, in Russia i 599
Manustupration as a cause of insanity vii 29, 54
Map, medical, of continent, desirable i 500
Marasmus, brain of children in . xxi 390
deaths from, in Ireland . . xviii 40
of the muscles . . . xxi 372
MARC, M., his jurisprudence of insanity x 129, 164
MARCARD on the waters of Pyrmont xiv 319
MARCHAL, M., on pelvic abscesses . xix 559
MARCHAND, Dr., on urea . . viii 550
MARCHETTI, Dr., on ophthalmoscopy, &c. v 31, 36
on the Tuscan maremma . . xxi 205
March of a regiment in India, effects of xiii 348
Marching in India, three rules for . xxiii 343
Mare, intestinal strangulation in a . xii 538
Maremma of Tuscany, statistics of . xxi 205
Marienbad, chalybeate waters at . xiv 355
mineral springs at . . . xiv 344
Marigold, use of, in uterine disease xxi 472
Marine air, preservative from cold . xiii 390
invertebrata, temperature of . xii 531
MARINUS, Dr., his oration in Brussels i 606
MARKHAM, Dr., on Parisian surgery xi 218
Mark lane, human specimens in . viii 524
Maroon town, Jamaica, salubrity of viii 216
Marriage, considerations on . . x 518
Dr. Ryan's book on . . v 442

Marriage, early, Dr. Loudon on . xvi 230
in relation to insanity . . vii 27
Marriages, early, on prohibition of. iii 474
evils of early, to offspring . iii 473
in different seasons . . xviii 202
MARRYAT, Dr., his Art of Healing quoted x 541
on large doses of emetic tartar, in 1788 . . . . . i 416
MARSDEN, Dr., of Nottingham, his death i 615
MARSHALL, Dr., on spinal irritation i 459
MARSHALL, Mr., his Military Miscellany xxiii 163
on soldiers . . viii 537, xviii 117
Marsh fever, observations on . . xiii 350
treatment of, cases of . xxiii 266
fevers, M. Boudin on . . xiv 43
treatment of . . . xiv 47
maladies in the blood . . xiv 46
malaria, on the nature of . xiii 351
miasm, adverse to phthisis . xiv 44
Marshes, agency of, in causing fever viii 216
artificial, in China, fever from. xxii 181
in Tuscany, on fever in . . xxi 208
salt, on malaria from . . xiii 350
MARSH, Dr., on inflamed epiglottis vi 261
MARSH, Mr., his apparatus for arsenic iv 360, xiii 190
froth in his apparatus . . xi 48, 39
his test for arsenic, objections to xx 329
modifications of his apparatus . xiii 191
MARTIGNY on pulmonary exhalation i 242
on watery vapour from the lungs i 242
MARTIN, General, his case of lithotrity xii 404
MARTIN, M., on education of mothers xiv 527
on pregnancy . . . viii 37, 50
MARTIN, Mr. J. R., on epidemics in Calcutta . . . . xvi 206
on tropical climates . xiii 347, 364
MARTIN, Mr. P., his translation of Louis i 400
MARTIN, the incendiary x 141, xv 87, 93, 274
MARTINET, error in his pathology . iii 68
MARTINEAU, Miss, case of clairvoyance by . . . . . xix 462
her Essays . . . . . xviii 472
on mesmerism . . . xix 429
Martinique, on phthisis at . . xviii 158
MARX, Professor, estimate of his works xix 126
German adieu to . . . xv 29
his Akesios . . . . . xix 121
letters to dead friends . xix 123
merits and works . . xiv 147
recollections of England . xv 19
tribute to Stieglitz . . xxiii 544
on Dr. Abercrombie . . xv 20
Dr. John Thompson . xv 20
Herophilus . . xv 106, 112
Paracelsus . . . xiv 147
pathology and therapeutics xvi 472, 476
the effects of civilization xviii 237
various pathologies . . xvi 473
Maryland, (U. S.) University of . ii 587
Masters in surgery, history of . xiv 405
the four, their method with divided intestine . . xxiii 21
MASSALIEN, Dr., on the facial nerves vi 200
Mastication, digestive effects of . xxiii 393
Mastitis puerperalis, use of ice in . xiii 108
Masturbation, observations on . xv 360, 362
preventives of . . xv 360, 362
symptoms from . . . xv 346, 362
Masulipatam and Kamptee, account of xxiii 109
MATEER, Dr., on dropsy . . iii 548
Material ideas, action of, on the brain xxiv 192
on the nerves xxiv 193
Unzer's doctrine of . xxiv 186
Materialism, remarks of Dr. Daubeny on xi 136
Materia Medica, classification of xvi 189, xvii 243
compendiums of . . ix 541
condition of . . . xvi 155
Dr. Bellingham's . . xiii 521
Dr. Dierbach on the . viii 353
Dr. Dunglison's . . xvii 242
Dr. Neligan's . . . xvii 529
Dr. Paine's work on . xiv 213
Dr. Pereira on viii 353, xvi 352, 362
Dr. Royle's Manual of . xxiii 244
Dr. Thomson's . xvi 350, 352
Dr. Ure's compendium of vi 516
Mitscherlich on the . viii 353
museum of, in Edinburgh i 307
study of . . . . i 307
syllabus of, by Dr. Johnstone i 193
Materia peccans in disease . . xxiv 319
Materies morbi in contagions . xxiii 315
Maternal imagination, effect of, on fœtus . . . . xix 369
Maternité at Cairo . . . vi 593
at Paris, account of . . i 281
Mathematical basis in medicine . vi 576
study, disadvantages of . . ix 420
utility of . . ix 412, 420
Mathematicians, longevity of. . i 344
Mathematics, importance of the study of . . . . xxiv 219
in medical science . . . iii 40
in relation to medicine . . xiii 257
Matico, styptic properties of . . xvii 100
Matlock, tepid water of . . xiv 352
Matter, on the infinite divisibility of xi 136-39
on the nature of . . . xxi 120
MATTEUCCI, Professor, on physical phenomena . . . xxiii 377
MATTHEW, Father, beyond all praise xvii 53
MAUNOIR, M., on cataract . . vi 50
MAUNSELL, Dr., on children's diseases iii 439, xi 198, 210
on political medicine . . viii 547
Mauritius, calculous diseases in . xvii 288
diseases and mortality at . xi 379
endemic hematuria in . . xv 481
mortality of black troops at . xi 381
topography and climate of . xi 379
MAUTHNER, Dr., on diseases of children xxi 381
MAXFIELD, Mr., on ulcers of the legs xv 226
Maxillary bone, superior, extirpation of xviii 340
upper, removal of vii 250, xx 195
MAXWELL, Dr., on yaws and tetanus ix 531

May, Mr., on ulceration of the brain viii 275
Mayer, Dr., his theory of organized bodies ix 525
on ciliary motions . . . iii 465
the brain and nerves . . i 556
the umbilical cord . . xix 546
Maygrier, M., his anatomy . . ix 538
Mayo, Dr. T., on insanity . . xxiv 225
on impunity of the insane . xix 33
Mayo, Mr., a pupil of Sir C. Bell . ix 135
dogmatism and contradictions of xiv 526
effect of hydropathy on . . xxii 453
his claims of discovery . . ix 135-37
claims to distinction . . v 536
Human Pathology . . iii 1, 55
opposition to Sir C. Bell . ix 136
Outlines of Pathology . iii 301
Philosophy of Living . v 380, 383
physiology . . . v 75, 100
objections to plan of his work iii 55
on diseases of the brain . . iii 302
hydrocephalus . . . iv 246
mesmerism . . . vii 349
omissions in his work . iii 63
syphilis . . . x 381, 419
the cold-water cure . . xxii 428
the nervous system . . xiv 525
the roots of the nerves . v 486, 533
ulceration of cartilage . ii 163
prejudice of . . . . xiv 526
remarks on his Outlines . . iii 97
Mayor, M., his surgical eccentricities xx 461
Mayow first truly explains respiration i 383
Meade, Mr., on fractures . . xi 463
Meals, habits in reference to . . xvii 46
proper intervals between . ii 380
proper times for . . . xvi 220
Roman, account of . . . xvi 220
Mean result of physiological acts . iii 41
Measles, and scarlatina, Dr. Burrowes on xii 98
treatment of . xv 382
child born with . . . xx 549
Dr. Mackintosh on . . iii 108
false, without catarrh . . xxi 467
in America . . . . xxi 364
incorrectness of, M. Billard on xi 203
inoculation of . . xx 211, xxi 364
more than once, cases of . ix 442
report on xvii 561, xx 559, xxiii 306
results of inoculation of . . xv 235
secondary, in a child . . vii 147
stomatitis complicating . . xvii 562
Measuring, medical, on the art of . xx 311
Meat, boiled and roasted, tenderness of xxiv 271
on zinc plates, remains long fresh ii 397
salted, Liebig on . . . xxiv 272
Meatus urinarius, female, removal of viii 587
finding the . i 115
Mechanical power in organism, source of . . . xiv 499, 502
Meckel, M., his Manual of Anatomy iv 497
on the corpus luteum . . iv 462
Meconine, its qualities . . . ii 207
Meconium, chemistry of . . xvii 436
Meconium, composition of . . xx 93
constituents of . . . xix 592
Medals, prize, of the Royal Society iii 291
Median race, ancient, or Persian stock xxiv 457
Mediastinum, on cancer of . . xiv 243
Mediate agency, Dr. Prout's idea of xxi 121
Medical academy, Dr. Gunther on a i 192
of St. Petersburg . iv 268, 272
annals, Austrian, German . i 219
anthology, Italian . . . i 222
art, advantages and benefits of iii 200
ancient dignity of . . xix 122
imperfection of . . iii 200
aristocracy, fancies of . . xiv 407
association of Prussia, in Berlin ii 582
associations in America . . iii 273
authors, Hindu, account of . xxiii 526
living, lexicon of . . xvii 504
misdoings of . . . xxi 499
old, taste for . . . xv 28
bibliography, by Dr. Forbes . i 513
bill, new, for Norway . . xix 188
Sir J. Graham's . . xix 193
alterations in xix 549
board of examination in Prussia i 301
books, remarks on writers of . iii 355
cases, treated by mesmerism . xxi 478
civil functionaries in Prussia . ii 302
college in London, scheme of a xiii 268
colleges, multiplication of . xiv 411
corporations, British statistics of i 306
wretched doctrines of . xiv 408
degrees in Belgium . . iv 282
scheme proposed for . x 203
deontology . . . . xxi 438
diploma in Paris, examinations for i 495
doctorate in Prussia, requisites for i 296
edict, ancient Indian . xxiii 522, 523
education . . . . xix 194
documents on . . viii 287-94
principles of . . . x 175
remarks on . . . i 2
Sir B. Brodie on . . xxiii 240
Essays, by Dr. H. Sealy . . iv 196
of St. Petersburg . . xxi 460
ethics in the United States . iii 276
etiquette, Mr. Banks on . . x 215
evidence, Dr. Holland on . viii 483
in infanticide . . . vii 139
remarks on . . . vi 122
examination in Russia i 600, iv 270, 278
Examiner, of Philadelphia . vi 260
experience, Dr. Cowan on . i 170
faculties of universities . . ii 577
faculty, scheme of one . . xiv 410
graduations, Edinburgh, 1836 iii 292
habits in chronic diseases . xxii 454
history and theory, chairs of . xxiii 106
humility, observations on . xxii 161
institutions, foreign . . i 289
German . . . . i 499
in America . . . iii 273
of the continent . . i 494

Medical journal, new quarterly . xxiv 598

journals, American . . i 228

British, list of . . i 223

Danish . . . . . i 227

French . . . . . i 223

German . . . . . i 217

Italian . . . . . i 221

jurisprudence, by Dr. J. B. Beck iv 87

cases in . . . . . vii 454

Dr. Most on . . . . vii 530

Dr. Traill's . . . . iii 492

education in . . . . x 196

in England . . . . ix 54

Manual of . . . . xvi 68

M. Bayard on . . . xii 174

Mr. Hoffman on . . iv 489

Mr. Taylor on . ii 390, 422

term improper . . ii 422

jurist, duty of . . . . v 172

qualifications of . . x 197

jurists, recommended . . ii 395

lecturers in Prussia, number of i 296

Lexicon, Dr. Dunglison's . viii 548

life, moral aspects of . . xxiii 467

literature, of Russia in 1770 . i 606

of the present day . . xxiii 37

professional neglect of . xxiv 592

man, on the skilful and ignorant xvii 189

men, between capital and labour xiv 460

duties and rights of . xxi 438

estimation of, abroad . iii 452

literary habits in . . xxii 120

of Prussia, their classification i 300

on mere practical . . iv 84

partakers of their own medicines i 260

scientific pursuits of . v 317

secret of success of . . xvii 191

tables of mortality of 850 i 202

term of life of . . i 200

military, education in Prussia . i 297

Notes and Reflections . . viii 482

observation, remarks on . . xxiii 104

observer, a lenient judge of men v 413

officer of inquiry for towns xxi 346, 349

officers, public, remarks on . xv 345

organization, plan of . . xxiii 105

panorama . . . . . xxiii 86

papers in British journals . xi 534

philosophy, Dr. Bouillaud on . iii 384

police, observations on . . xv 329

of the United Kingdom . xxiii 86

plan of . . . . xv 342, 345

treatises on . . . . xiv 446

Portrait Gallery . . . . vi 194

practice, general and special . x 200

influence of theory on . ii 5

radical fault of . . xxi 516

reflections on . . . xiv 423

state of French . . i 495

subdivision of . . . x 200

practitioner, qualifications of . xxiv 221

practitioners, privileges of . xvii 123

protection of . . xix 222-31

Medical problems, by Dr. Griffin . iv 252

by Messrs. Griffin . . xxi 92

profession, Dr. Watson on the xvii 122

fusion of grades in . . xiv 410

in England . . . xiv 409

Germany . . . xiv 409

Prussia . . . i 301

Russia . . . iv 263

modern divisions of the . xiv 405

proposed new organization of xxi 531

respect for the . . iii 201

Sir B. Brodie on the . xvii 192

social duty of the . . xiv 459

relations of the . xiv 460

source of evils in the . xiv 409

professors in Prussia, number of i 296

reform . . . ix 281, x 297

documents on . . viii 294-9

Dr. von Walther on . xiv 402

in Spain . . . . i 608

memorial on . . . xiii 580

petition for . . . viii 612

Sir James Clark on xiv 296, xv 529

works on . . . xix 193

relief under New Poor Law . vi 586

Repository, general, German . i 220

research, nature and aim of . vii 115

researches by Dr. Harlan . ii 518

Review of Rio Janeiro . . ii 234

scepticism, remarks on . . xxiii 469

school, at Bologna . . i 498

of Birmingham . . i 207

Breslau . . . i 299

Griefswald . . . i 299

Magdeburg . . i 299

Manchester . . i 206

Munster . . . i 299

schools, in Russia . . . iv 268

introductory lectures at . i 203

of the ancients . . xvii 457

science, based on observation . xii 10

departments of . . xxiv 294

disadvantages of . . xii 11

early history of . . xvii 450

essay on the nature of . xviii 235

imperfection of . . xii 3, 8, 20

low state of . . . vi 99

philosophy of . . . xx 140

slow advance of . vii 115, 116

use of philosophy in . i 368

sects of Germany . . . xvi 472

services, nobleness of all . xiv 408

societies, in London . . viii 535

in Prussia . . . ii 577

remarks on . . . ii 577

society, at Munster . . ii 581

of observation . . v 154

staff of the queen . . . iv 568

statistics, Dr. Casper on . xxiv 91

principles of . . . xii 1

Student, Dr. Dunglison's . iv 496

students, in Bavaria . . iv 362

in Prussia, number of . i 296

Medical students in the United States . ii 593

in London, remarks on . ii 473

studies, moral effect of . . xxi 441

results of . xix 122

Sir B. Brodie on . . xxii 160

study, Dr. Symonds on . . iii 201

on education previous to . ii 474

order of, at Bologna . i 499

proper course of . . x 179

systems, influence of, on mortality x 423

teachers, responsibilities of . xvii 122

Terms, Dictionary of . . ii 508

theories, observations on . xxi 498

treatment, efficacy of . . xxii 561

vocabulary, a useful one . iii 206

witnesses' Bill . . . ii 607

remarks on . . . vi 122

works for general readers . xi 152

Medication, on external and internal vii 71

Medici, Dr., on the structure of bones i 549

Medicinal actions, by nervous conduction xiv 142

through circulation . xiv 143

two sorts of . . xiv 142

agency and nature . . xii 88

agents, Liebig on . . xiv 516, 519

prescriptions, four parts of . xvi 203

Medicina perturbatrix, mischief of . v 567

Medicine, a composite science . vii 115

advancement of . . . xxi 514

ancient Indian . . . xxiii 521

and surgery, anciently united . xiv 406

Dr. Morand on . . xxiii 372

one science . . . vi 98

on reuniting . . . xiv 403

relations of . . . xiv 402

an estimative science . . xxi 524

application of, to science . xiv 460

bad effects of continued . . xxii 170

before Hippocrates' time . xvii 459

Bonaparte's opinion of . . ix 478

cause of the many systems of . xvii 472

causes of small progress in . xxiv 291

clinical, Dr. Latham on . . ii 473

in Italian hospitals xxiv 360, 363

distressing uncertainty in . vi 110, 111

doses of, observations on . xv 203

Dr. Alison's Practice of xv 533, xviii 213

Dr. Billing's Principles of . v 200

Dr. Dunglison's Practice of . xiv 220

Dr. M. Hall's observations on xxiii 185

Dr. Tweedie's Library of . x 231

Dr. Williams's Elements of v 353, xiii 89

Principles of . . xvii 472, 502

Eastern, history of . . xxiii 522

ethical relations of . . . xix 121

empirical, advance of . . vi 110

method in . . . xxiv 290

etherization in . . . xxiii 566

facts wanted in . . . xii 21

false facts the bane of . . vii 117

foreign, reports of . . . i 289

French school of . . . iii 384

glorious aim and end of . . xviii 255

Medicine, great defect in the study of xxi 509

hardly a scientific art . . vi 110

Hindoo, account of . . vi 506

hypothetical, Dr. Lanza on . xxiii 40

improvement of . . . vi 196

in Prussia, state of . . i 289

in Russia, history of . . i 597

is it a science? . . . vi 103

its difficulties . . . vii 116

improving character . . iv 285

progress and prospects . xxii 235

relation to chemistry . xvi 145

legal, treatises on . . ii 390

'l'idée mère' deficient in . . vi 294

mathematical basis in . . vi 576

meet example of . . . xii 21

mischief of false principles in ii 4

narrow basis of its rules . vi 110

neglect of, by students . . ii 473

new researches in . . . xx 340

obstacles to advancement of . xxiii 62

philosophical treatise on . x 55

philosophy of . . . xxiii 39

positive, Dr. Lanza on . . xxiii 37

foundation of . . . xxiii 45

new requisites for . . xxiii 63

power and benefits of . iii 200

practical, Dr. Cowan on . . xx 509

Dr. Macrobin on . . i 203, 207

practical education in . . x 195

practice of, based on theory . vi 110

Dr. Craigie's . . . iii 452

Dr. Dunglison's . . . xviii 285

Drs. Bright and Addison . iii 452

in Royal Navy . . xxiii 107

present state of . . xxi 258

present progress of . . iii 160

progression and proficience of vii 117

progress of, report on . . xvi 268

systems of . . vi 294

regeneration of . . . xxi 261

rational empiricism in . . vii 111

method in . . . xxiv 290

relation of to surgery . . xvi 507

remarks on the progress of . xix 21

requisite test in . . . xii 21

retrospect of . . . . xi 224

Rousseau's apophthegm on . xii 22

science of, definition of . . xii 2

sciences auxiliary to . . xii 2

specific, new system of . xxii 565, 567

state of, in Austria . . i 1

in Denmark . . ii 285, iii 565

France, &c. . . iii 447

Italy . . . . iii 576

Russia . . . iv 263

the United States . iii 273

the art of, reflections on . . xxii 189

language necessary in . xii 21

most difficult science . xii 10

uncertainty of . . . xxiii 40

value of systems in . . xix 371

veterinary and human . . xxiv 82

Medicine, when an art merely . xii 1

a science . . xii 1

works on the practice of . ix 461

Medicines, acting primarily on stomach xvi 194

action of, on nervous centres . xiv 144

on organs . . . xiv 145

on the blood . . . viii 355

Rognetta on . . . xxi 160

and the blood, reaction of . xiv 143

affording eligible form for . xvi 202

circulating in the blood . . xiv 144

comparative use of . . iii 269

disuse of . . . . . xxii 237

effect of innervation on . . xiv 145

for merchant ships, list of . xxiv 213

high prices of, in Bengal . xvi 353

homœopathic, mode of prescribing xxi 236

impressibility of organs by . xiv 145

not decomposed in transitu . xvi 194

of false celebrity . . . xiii 55

on practitioners keeping . xxi 525

operation of certain . . xiv 517

prices of, in America . . iii 282

primary effects of . . . xiv 146

remarks on the action of . iii 223

secondary effects of . . xiv 146

solubility of, in primæ viæ . xvi 201

surfaces adapted for . . xiv 145

table of new and old names of iv 116

their uses, &c. . . . . xvii 529

Medico-chirurgical academy for the army in Prussia . . i 298

anatomy, treatise on . . xx 133

provincial schools in Prussia . i 299

Sciences, Journal of . . i 223

Transactions ii 158, iv 138, vii 140, xi 447, xiv 49, xv 147, xviii 364, xx 83, xxiii 410, xxiv 321

Medico-legal reports, remarks on . iii 248

Medico-statistical details, on age in iii 270

Mediterranean, and Peninsular naval command . . . . . xi 193

command, diseases on the . xvii 319

liver complaints in . . . ix 65

naval station, mortality in . xv 8

Medulla, hypertrophy of . . xvi 382

red and inflamed in osteitis . ii 537

Medulla oblongata, anatomy of xi 542, xviii 427

Dr. Stilling on . . xviii 134

functions of iv 486, xvi 367, xviii 138

Mayo and Solly on . . v 106

Mr. Mayo on the . . v 534

Mr. Solly on . . . xxiv 574

respiratory nerves in . xviii 136

structure of . . . xviii 135

true limits of . . . xviii 134

spinalis, dependent life of . i 558

Mr. Hilton on . . vii 277

Medullary cancer, probably scrofulous ii 20

fibres, anastomosis of . . vi 215

primitive, form of . . vii 541

fungus, distinct from cancer . i 471

Dr. Baring on . . i 469

Medullary tumour, constituents of xvii 446

Megatherium, classification of the . x 211

Megatheroids, fossil, dental structure of . . . . . xxi 73

Méglin's pills, composition of . xvii 159

Mehliss, Dr., on diseases of the diaphragm . . . . xxiv 166

on virilescence, &c. . . vi 75

Meibomian glands, Dr. Zeis on the v 218

enlargement of . . xi 76

in mammalia . . . vi 514

Meigs, Dr., his translation of Colombat xxi 83

his treatise on midwifery . viii 37

Melancholia, opiates in . . . xxiv 150

causes of . . . . vii 26, 27

Dr. Seymour's success in . xxiv 151

travelling injurious in . . xxiv 153

Melancholic temperament, remarks on vii 26

Melancholy, actors very subject to . vii 26

in children, cause of . . vii 26

professions subject to . . vii 26

Melanism, complete and partial . viii 20

Melanoma, Dr. Carswell on . . i 135

Melanosis of the kidneys . . xv 491

remarks of Dr. Walshe on . xi 163

Melanotic deposit, constituents of . xvii 446

pseudo-, formations . . i 135

structures, in sputa . . xvii 521

Melchior, M., on strabismus . xi 474, 491

Melicher, Dr., on congenital luxations xxi 393

Melier, M., on provisions, &c. . xviii 155

on quinine in intermittents . xviii 162

Melituria, or diabetes mellitus . vii 164

pathology of, facts in . . vii 164, 165

sugar in the blood in . . vii 164

secretions in . vii 165

use of magnesia in . . . vii 165

Mellon, a chemical radical compound xi 133

Melolontha vulgaris, description and

*plate of* . . . . . i 539

Meloplastic, etymology of . . vii 408

operation . . . . . vii 410

operations, Dieffenbach on . xxi 305

Melzer, Dr., his history of foundlings xxii 338

Membrana decidua, situation of . x 587

pigmenti, report on . . xxii 285

tympani, description of the . viii 74

diseases of . . . iii 90

hearing with perforated . iii 91

inflammation of . . iii 92

laceration of . . iii 91

morbid states of . . xxiv 28

perforation of. iii 91, 93, xxiv 36

relaxation of . . . iii 90

structure of . . . viii 73

use of . . . . . viii 84

Membrane, false, ingredients of . xvii 444

germinal . . . . . xx 290

secreting mucus, remark on . xx 124

Membranes, adhesion of serous . vi 232

animal, as filters . . . xix 256

endosmose in various . . xxiii 387

false, Dr. Hodgkin on . . iv 36-50

Membranes, in thorax, Gendrin on . iv 44

organization of . . iv 38

structure of . . . xv 234

in labour, on rupturing the . iii 414

on the nerves in . . . xxi 546

ovarian, structure, &c., of . xvii 272

reflected, erysipelas of . . ii 24

rigidity of, in labour . . iv 402

serous, Dr. Hodgkin on . . iv 31

gangrene in . . . iv 52

no distinct . . . xviii 80

osseous deposits in . . iv 53

tubercles in . . . iv 53

ulceration of . . . iv 51

ultimate structure of . iv 32

synovial, diseases of . . x 331

Membranous labyrinth of the ear . viii 80

Memoir of Dr. Andrew Combe . xxiv 581

Memoirs, French, military, medical xxiii 74

of the Bologna Society . . v 167

Parisian Society . v 154, vi 28

Royal Academy of Medicine . xviii 152, xxi 408

Society of Observation . xix 390

Memory, curious case of loss of . iv 478

in relation to insanity . . xv 419

nature of . . . . xxiv 191

of idiots, education of . . xxiv 12

of words, cases of loss of . iii 261

MENECRATES and Philip of Macedon xix 122

MENGHINI, his doses of cream of tartar iii 432

Men, literary, disorders of . . iii 197

races of, physical alterations in xxiv 57

identity of . xv 182

physiological identity of xv 183

psychical and moral identity of . . xv 183

Meningeal apoplexy, Dr. Prus on . xxi 409

artery, on rupture of . . xv 173

inflammation, bellows-sound in i 485

Dr. Neisser on . . xxii 384

Meninges, cerebral, cancer of . xxii 310

Meningitis cerebralis with emphysema xxii 460

and cerebritis, diagnosis of . xii 101

cerebro-spinal, epidemic xv 236, xx 256

Dr. Hope on . . . . xii 101

epidemic, in France xviii 160, xxiii 82

granular . . . . ix 218

in children . . . xvi 445

infantile, symptoms of . . xxiv 41

treatment of . . . xxiv 42

mesencephalica in children . xvii 552

spinal, sporadic . . . xx 257

tubercular, in children . . xvi 445

Menorrhagia, Dr. Churchill on . vi 92

ergot of rye in . iv 369, vi 92, x 44

gallic acid in . . . . xvii 542

juice of the nettle in . . xx 539

opium in . . . . . x 81

report on . . . . . xxiii 292

resemblance of abortion to . vi 84

Menses, cessation of . . . xix 86

effect on health . iii 397

effect of temperature on . . xix 85

Menses, influence of, on diseases . xiv 390

only during pregnancy, cases of iv 451

suppression of, blue skin in . xiv 392

tables of . . . . . i 103

Menstrual blood injurious . . ix 545

characters of . x 257

the same as arterial xiv 387

discharge, observations on . xiv 388

analysis of . . xvi 228

fluid, analysis of . . . xiv 387

source of . . . vii 233

period, on duration of . xiv 385, 388

periods, statistics of . . xviii 177

punishment of male Jews . vi 79

secretion, analysis of . . vi 446

Menstruation, after parturition . xiv 386

age in relation to . . . xiv 384

and œstrum, Dr. Davis on . iii 398

and its diseases . . . x 67

bloodletting in . . . xiii 404

cases of death during . . x 68

decline of, diseases of . . xix 347

difficult, bougies in . . iii 113

discharge of ova in . . xix 84

discharges vicarious of . . xx 540

disorders of . . . . vi 92

report on . xvii 542, xxiii 291

Dr. Clutterbuck's idea of . v 187

Dr. Davis on . . . . iii 397

Dr. Laycock on . . . xii 63

Dr. Power's theory of . . viii 531

during pregnancy . . . v 577

earlier in towns . . . xiv 385

expulsion of ova in . . viii 530

fever prior to . . . iii 351

from irritation of nipple . . xiv 395

function of ovaries in . . x 592

Gendrin's theory of . . x 68-71

imperfect . . . . . x 76

in acute diseases . . . x 76

in advanced life . . . iii 397

in a young child . . . xi 225

in conception . . . xiv 386

indecent neglect in . . xxi 84

in different places . . . xvii 276

influence of on milk . . xvii 274

in old age . . . v 256, vi 80

in pregnancy, not rare . . iv 451

inquiries respecting . . xiv 391

in relation to pregnancy . iii 140

insanity in relation to . . xiv 395

in the male . . . . vi 79

later in the country . . xiv 385

milk more serous by . . xvii 443

Mr. Jones's theory of . . viii 530

of prostitutes . . . iv 69

on risk at its cessation . . iii 398

pathology of . . . . xiv 391

period of, in France . . xx 138

phthisis in relation to . . xiv 395

premature . . . . . xix 86

report on . . . . . xxii 289

returning in old age . . x 560

serotine, remarks on . . iii 397

H Robertson

Menstruation, state of ovaria in . x 68, 70

state of uterus during . . viii 39

statistics of . . . . . xiv 382

theories of . . . . . xix 84

the ova in relation to . . xvii 271

vicarious, case of . . . xiv 394

death from . . . xiv 395

from lungs . . . xiv 395

remarks on . . . xiv 395

singular case of . . xi 179

Menstruous fætor, an emmenagogue vii 234

Mensuration of thorax, in auscultation i 480

Mental acquirements of medical men xxi 443

actions, changed into reflex . xxiv 204

affections, rotatory motion in . xv 72

attention, effects of on organs. viii 492

cultivation in regard to health iv 60

decay, signs of . . . . xv 420

derangement, Dr. Moreau on . xxiii 217

Dr. Seymour on . . xxiv 150

disease, hereditary . . . xiii 259

diseases, Dr. Webster on . xxiii 414

disorder, management in . xxi 463

dynamics, Mr. Green on . xxiv 216

emotion, effects of, on the brain iv 437

excitants, Dr. Thomson on . xvi 361

excitement in regard to health iv 60

exercise, rules for by Dr. A. Combe i 337

functions, remarks on . . xiii 477

incapacity, partial, remarks on vi 127

maladies, M. Esquirol on . vii 1, 4

treatise on . . . . xx 520

physician, the art of the . ix 189

powers of man and animals . xi 101

precocity, Dr. Brigham on . iv 61

Mephitis and miasm, puerperal . xiii 102

Merat, M., on French mineral waters xiv 309

Mercer, Dr., his plagiarisms . xi 222

on the organs of hearing . xi 222

Mercier, Dr., on the prostate gland ix 260

Mercurial cachexia, bath for . . iv 254

example of . . ii 21

signs of . . . ii 21

remarks on . . ii 22

erythema and erethismus . v 23

fætor, cause of . . . . v 139

inunction, in puerperal fever . ii 93

inunctions, use of large . . viii 419

ointment, new mode of preparing xi 254

pills of . . . . . xxiii 365

paralysis, remarks on . . . x 449

pills, for the syphilides . . xxiii 365

ptyalism, composition of . viii 346

salivation, in dysentery . . xxiii 151

sore mouth, treatment of . xvii 125

stomatitis, the blood in . . xvii 145

Mercurialization, in amenorrhea . iii 399, 400

rapid, in phthisis . . . xvi 253

Mercurials with narcotics . . xvi 200

*Mercury*, head of, on expression in xviii 447

Mercury, action of, in inflammations xx 190

in syphilis . . . v 22

on the liver . . viii 361

alone, cure of iritis by . . ii 348

Mercury, and antimony, remarks on ii 145

as a poison, detection of . xx 332

a substitute for repeated bleeding i 399

best mode of administering . xxii 169

bichloride of, great utility of . xvi 271

biniodide of, ointments of xiii 75, xix 518

binoxide of, solubility of . viii 361

continuance of, in syphilis . v 24

curative effects of . . . xvii 125

diseases from abuse of . . xi 342

dry course of, in syphilis . v 23

effect of injecting, on lungs . viii 323

effect of small doses of . . vii 581

effects of abuse of . . ix 240

employed in Pliny's time . vi 508

external use of, in smallpox . vii 552

formation of tubercles by . viii 323

idiosyncrasies in regard to . v 23

iodide of, observations on . xiii 74

increased use of, in France . xii 89

in diseases of the eye . . x 8

dysentery, Mr. Twining on . iii 332

fever in India, remarks on . xxiii 118

hepatic affections, Dr. Budd on xxi 48

hypertrophy of heart . . vi 388

indurated testicle . . i 476

inflammation, Dr. Alison on xiii 338

Dr. Watson on . . xvii 125

Mr. Miller on . . xix 527

of brain . . . xii 102

pericarditis . . . xx 192

phthisis . . . . . xix 145

scrofula, remarks on . . xxiii 519

secondary syphilis, evils of . xix 522

small doses, effects of . . ii 145

smallpox and cowpox . . ix 249

in syphilis, Dr. Bene on . i 17

Dr. Colles on . . v 22

Mr. Carmichael on . x 381

M. Cazenave on . . xxiii 350

remarks on . . v 21

rules for its use . . v 22, 23

Sir C. Bell on . . vi 171

syphilitic eruptions . . v 25

yellow fever, Dr. Bone on . xxiii 428

liquid proto-nitrate of . . xv 36-7, 47

metallic, in introsusception . viii 422

mode of action of . . . xvii 125

Mr. Colles on the use of . vi 388

nodes, &c., from . . . v 131

on the protracted use of . v 23

preferred in the syphilides . xxiii 364

poisoning by . . . xviii 542

preparations of, for syphilides . xxiii 365

proto-iodide of, pills of . . xxiii 368

reprobated in splenalgia . . i 566

secondary symptoms from . ix 237

sign of its being too powerful . v 23

syphilitic symptoms from . v 131

therapeutic value of . . xii 88

use and abuse of . . x 245

use of, in acute inflammation . xii 102

chronic dysentery . iii 145

hepatic affections . xii 87

Mercury, use of, in neuralgia . . vi 573
venereal hectic . v 24
value of in inflammation . x 532
MÉRICOURT, Dr., on diseases of the
breast . . . . . xxii 156
Mesenteric artery, aneurism of . xiv 64
glands, cancer of, rare . . xxii 299
on disease of the . . ix 479
disease of . . . . xii 117
tubercular, in children . xvii 311
phthisis of children . . xvii 311
tumour, remarkable, case of . xiv 126
MESMER and Father Hell . . vii 306
and Mlle. Paradis . . . vii 307
and Mesmerism . . . vii 305
at Paris . . . . . vii 307
death of . . . . . vii 313
description of . . . . vii 313
his apparatus and process . vii 308
magnetising the sun . . vii 314
pecuniary affairs . . vii 310
Mesmeric aphorisms and axioms vii 315, 316
contradictions . . . . vii 330
diagnosis, Dr. Elliotson on . xx 287
doings, account of . . . xix 477
ecstatics, attributes of . . xix 448
facts, two classes of . . xix 431
insensibility to pain . . xix 436
lucidity, on the evidence for . xix 450
remarks on . . . xix 449, 469
medical cases, remarks on . xxii 483
sleep, example of . . . xix 433
operations during . . xix 434, 442
therapeutics, remarks . . xix 483
Mesmeriser, charming sensations of the xix 474
Mesmerising from a separate room xix 478
Mesmerism, aristocratic patronage of xxi 535
as a new art and science . xix 485
claim of, to fair trial . . xxii 485
community of sensation in . xix 480
Dr. Forbes on . . . xxi 277
explanation of . . . . xii 67
in Blackwood's Magazine . xix 485
England, 18th century . vii 312
India, Dr. Esdaile on . . xxii 475
relation to warmth . xix 480, 482
intuition in . . . . . xix 466
Mr. Walker's opinion of . xiii 211
objectivity of, remarks on . xix 479
on occult influence in . . xix 475
pathological nature of . . xix 474
prevision in . . . . . xix 468
reflections on . . . xix 483, 485
relation of hysteria to . xix 436, 447
review and estimate of . . xxii 486
simpler phenomena of . . xix 432
the imagination in . . . xix 475
theory of . . . . . vii 314
therapeutical action of . . xxii 478
the will in relation to . . vii 323-5
treatises on . . . . . xix 429
Mesocephale, Dr. Todd on the . xxi 457
Mesmerists, credulity of . . xix 478
Metabolic action of cells . . ix 523
Metabolic force in fermentation . ix 523
Metallic collyria, observations on . xxi 163
salts, therapeutic action of . xvi 154
tonic action of . . xi 442
sounds in chest, cause of . vii 569
tinkling . . . . . viii 451
cause of . . . . i 482
on the causes of . . xiii 536
Metals, as remedies, Dr. Kochlin on viii 353
poisonous, in air . . . xix 14
Metamorphoses, in the cirripedes . ii 214
supposed, in crustacea . . ii 214
Metamorphosis, hypothesis of, noticed i 392
of tissues, diversified . . xvii 40
Liebig on . . . xiv 511
remark on the term . . v 338
Metastasis, and sympathy, the same ii 8
from swelled testicle, case of . ii 9
in analogous structures . . ii 9
laws of . . . . . xxi 24
marked examples of . . ii 9
Mr. Travers on . . . ii 8
observations on . . . xxiv 319
purulent, Dr. Froriep on . i 569
Sig. Bellini on . . . xxiv 288
Metatarsal bones, luxation of. . vi 538
resection . . . . . xx 53
METCALFE, Dr., his caloric-theory,
remarks on . . . xviii 521
on caloric . . . xviii 513, 521
caloric, closing paragraphs of xviii 522
Meteorismus in fever, treatment of xv 466
Meteorological observations . . vii 294
Meteorology, and chemistry, connexion of xxi 118
and climate, treatises on . xx 215
Dr. Prout's treatise on . . xxi 109
obscurity of . . . . v 325
Methodist, medical sect . . xxiii 102
Methodus medendi, Dr. M'Cormac's xv 534
Methylene, a radical compound . xi 133
Metro-hemorrhage, Dr. Gendrin on x 77
Metrhymenitis, Dr. Kiwisch on . xiii 121, 122
Metropolitan convalescent institution xii 576
improvements, on the new . xix 381
Metropolis, suggestions on health of the xi 430
Metrorrhagia, remedy for . . xxi 89
Metroscope of M. Nauche . . iv 458
METTERNICH, Prince, and quarantine xxiv 244
MEYEN, Dr., on vegetable physiology x 248
MEYER, Dr., Kantian jargon of . xviii 146
on nervous fibrils xvii 379, xviii 134, 144
the Cæsarean section . . ii 568
Mexico, ancient civilization in . xxiv 475
Mezereon, ointment of . . . xiv 441
Miasm, observations on . . xi 447
fevers in London from . . xviii 503
Miasma, and contagion, Dr. Henle on ix 398
in newly-cleared countries . i 353
Miasmata, absorbed by the skin . i 331
in large towns, effects of . xxii 216
of towns, ill effects of . . xviii 502
organic, nature of . . . ix 399
Miasmatic atmosphere, fatal fevers in xviii 502
mortality in . . xviii 501

Michaelis, Dr., his cases of Cæsarean operation . . . . ii 270
on reposition of the funis . i 588
Michéa, Dr., on hypochondriasis xx 364, 375
Microcephalus in children . . xxi 390
Microscope, compound, first use of xiv 453
compression with the . . vi 397
Dr. Bennet on the . xiv 478, 481
Dr. Mandl on the . . xiv 478, 480
Mr. Paget on the . . . xv 542
remarks on the . . . vi 395
results from, in physiology . xiv 259
the most useful kind of . . vi 396, 403
the compound . . . vi 397
true estimate of the . . xiv 489
use of, in physiology . . v 333
Microscopical anatomy, history of . vi 397
illustrations . . . . x 550
investigations, Müller on . vi 424
observation, remarks on vi 395-7, 403
observations on the blood . xx 121
Microscopic Anatomy, Mr. Hassal's xxii 552, xxiii 252
remarks on . . ii 31
facts, on exclusive love of . xiv 489
investigation, defence of . xxiii 513
researches, popularity of . xiv 490
report, by Dr. Carpenter . xv 259
society, strictures on the . xiv 489
use of chromic acid . . xi 520
Microtomic compressor . . i 510
Midden-steads or dungheaps in towns xxi 340, 349
Middle ear, diseases of . . iii 97, viii 99
Middlemore, Mr., his flippancy, &c. ii 344
his inaccurate anatomy . . ii 351
on diseases of the eye . . ii 342
on the eye . . . . x 3, 22
Middlesex hospital, history of . xxi 282
lunatic asylum, report of . vii 2, 54
Midland Retreat, notice of the . i 308
Midwifery, and midwives, in France . i 495
British works on . . . xii 462
case of, Chinese . . . xiii 569
cases of, in the Maison d'Accouchement i 588
clinical, Dr. Lee's . . . xv 226
compendiums of . . . xix 58
deities, of, in ancient Rome . xiv 82
Dr. Bush's Berlin report of . v 576
Dr. Campbell's treatise on . xx 208
Dr. Collins's treatise on . . ii 71
Dr. Davis's system of . . iii 394
Dr. Hamilton on . . . iv 181, 398
Dr. J. Hamilton on . . iii 131
Dr. J. Reid's Manual of . i 512
Dr. Kilian's Operative . . iii 392
Dr. Lee's lectures on . . xvii 527
Dr. Meigs's treatise on . . viii 37
Dr. Ramsbotham on . . xv 527
Dr. Rigby's memoranda in . iii 494
Dr. West's report on xvii 533, xx 525, xxiii 277
effect of civilization on . . xviii 244
etherization in . . . xxiii 567
Midwifery, first printed book on . xiv 84
written rules for . xiv 81
French works on . . . xii 461
German works on . . . xii 461
history of . . . . xiv 80
first period of . . xiv 81
second period of . . xiv 81
third period of . . xiv 83
fourth period of . . xiv 83
fifth period of . . xiv 84
in ancient Greece . . . xiv 81
in Cairo . . . . vi 593
Mr. Ingleby's cases in . . iv 407
Mr. Rose's report on . . ix 220
new journal of, by Von Busch . i 545
of Abulkasem . . . xiv 84
Avicenna . . . . xiv 83
Hippocrates . . . xiv 81
Serapion and Ali-Abbas . xiv 83
the ancient Egyptians . . xiv 81
ancient Romans . . xiv 82
ancients . . . xiv 80
Arabs . . . . xiv 83
on showing instruments in . xiv 372
on various subjects in . . iv 398
operative, report on xvii 538, xxiii 283
Paul of Ægina on . . . xiv 83
plates of . . . ix 540, xiv 538
periods in the history of . . xiv 80
practical treatises on . xiv 366, xix 416
practice of, ungentlemanly . xiv 407
practitioners of, caution to . xiii 376
rules of, by Philumenos . . xiv 83
schools of, first Russian . . i 604
statistics, &c., report on xx 536, xxiii 291
treatises on . viii 37, 467, xii 461
use of reflex function in . . xvii 179
use of the stethoscope in . . v 596
works of Ætius of Amida, on . xiv 83
Midwives, Dr. Naegele's Manual for xix 58
on the employment of . . xix 59
Milan hospital, mortality in . . xxiv 379
Milan, on sudden deaths in . . x 420
tables of mortality in . . x 422, 426
topography and statistics of . x 421
Miliary fever, epidemic, account of . xx 233
Miliary, or sweating, epidemic fever vii 244
-pock, in the cow . . . ix 95
tubercle, nature of . . . xxiii 430
tubercles in serous tissue . . iv 54
Military, and naval medical schools . xvii 246
cooking, remarks on . . xxi 172
hygiene, remarks on . xix 382, 389
medical education in Prussia . i 297
guide books . . xix 377, 380
memoirs, French . . xxiii 74
officers of Prussia . . i 302
returns, remarks on . . xvi 34
prisons, diet in . . . xxiii 168
service, effect of civilization on xviii 243
punishments, Mr. Marshall on . xxiii 164
surgeon, practice of the . . xxiii 78
surgeons, authority of . . xviii 123-4
duties of . . . xviii 123

Military surgeons, instruction of . xix 378

surgery, cases in . . . iv 256

Dr. Ballingall on . . ix 243

outlines of . . . xx 202

Milk, abnormal secretion of . . xii 558

absorption of, by the lacteals . xxiii 497

adulteration of . . . xiii 79

analysis of cows' . . . xiii 78

and colostrum alkaline . . vi 184

pus, difference of globules of vi 185

vegetable diet in phthisis . v 189

whey diet, effects of . . v 565

case of absorption of . . ix 248

characteristics of good . . xiii 79

chemistry of the . . . x 127

composition of, stated . . vi 182

compounds which pass into . xvii 443

constituents of . . . xvii 443

curdled, restored by potass . xiii 79

difference of, in animals . . xxii 292

distaste for, by some nations . xvii 16

effect of medicines on the . x 126

passions on the . . x 126

elements of, in urine . . xii 326

in pregnancy xii 326

extemporaneous production of . xiv 229

fallacious appearance of . . vi 182

fatty substance of . . . xiv 222

human, globular character of . xviii 156

micrology of . . . xviii 155

richness in . . . xviii 156-7

three kinds of . . xviii 156

influence of menstruation on . xvii 274

in one breast salt . . . xii 431

in the breast of males . . vi 79

masses of, in the stomach . xix 29

microscopic characters of . xii 263

constituents of . . xi 228

mixture of, with pus . . vi 185

more serous by menstruation . xvii 443

mucous state of, and colostrum vi 183

nurses', statistical tables of . xviii 156

nutritive qualities of . . xvii 27

observations on . . . xvii 28, 443

of cows, effects of food on . xvii 25

of diseased cows, remarks on . xvii 25

nurses, remarks on . xvii 25

of nurses, examination of vi 182, xviii 155

may be too nutritious . vi 185

pathological changes in . . vi 185

purgative quality of the first . iii 441

remedy for increasing the flow of v 563

secretion and course of the . x 117

secretion of, and suckling . x 124

by aged females . vi 80

in suppressed menses xxii 156

supply of, in woman . . x 125

test of the richness of . . vi 185

the original food of man . . xxiii 496

Milk-abscess, and milk-fever, Dr. Hall on xxiii 198

benefit from ice in . . xiii 108

on early opening of . . xxii 157

Milk-cells of the mammary gland . x 116

Milk-globules . . . . . vi 182

chemical tests of . . vi 185

dissolved by ether . . vi 182

Milk-leg, observation on . . xxi 92

Milk-sickness, affects man and beast iii 218

euphorbia ipecacuanha in . iii 219

fatal case of . . . iii 218

in America, account of . iii 218

locality of . . . iii 218

prevention of . . . iii 219

treatment of . . . iii 219

Milk-teeth, absorption of . . ix 444

Milk-thrush, or muguet . . vii 82

Milks, different, composition of . xxiii 497

Milky-looking urine, cause of . . xi 343

treatment of . xi 344

Milky serum in diseases . . xx 74

Millepedes discharged from the stomach xiv 572

Miller, Prof., his Surgery . xix 524, xxi 488

on pictorial anatomy . . xiv 528

Milliners, their unhappy and diseased state i 366

Millengen, Dr., inconsistencies of xi 116

on treatment of lunatics . . xi 104, 116

specimens of his style . . xi 121

Minden, hospital ship, account of . xxii 179

dysentery in . xxiv 358

Mind, absence of, in excess . . xxiii 235

activity and supremacy of . xiv 310

and body, reciprocal influence of xv 413

and soul, ancient ideas of . xv 442

disorders of, on medicines in . xxi 462

Dr. Wigan on duality of . . xx 1

duality of, phenomena like . xx 16

influence of, on body . . iii 264

in relation to muscles . . xix 575

its power over disease . . viii 494

its relation to dyspepsia . . xii 274

mastering disease, Kant on . i 191

minor diseases of the . . xviii 472

the brain the organ of . . ix 197

unsoundness of . . . x 129

Mineral poisons, Dr. Prater on . xxii 545

springs of England, Mr. Lee on xiv 309

waters, action of . . . xiv 322-5

active ingredients in . xiv 322

alkaline, M. Petit on . xiv 350

and baths in gout . . xiv 333

carbonic acid in . . xiv 326

congestion of head from . xiv 340

diet when taking . . xiv 358

diseases benefited by . xiv 327

division of . . . xiv 325

effects of, undervalued . iii 203

French, M. Merat on . xiv 309

hot alkaline . . . xiv 347

hot saline . . . xiv 346

hot sulphurous . . xiv 346

in scrofula, facts on . xxii 153

Mr. Lee on . . . iii 202

observations on . . v 398

rules for drinking . . xiv 357

saline . . . . xiv 342

theory on . . xiv 332

scepticism on . . . xiv 321

Mineral waters, use of chalybeate . xiv 338
value of, in diseases . xiv 315
various treatises on . xiv 309-20
virtues of . . . . xiv 322
when especially applicable xiv 328
when inapplicable . . xiv 328
Miners, and sappers, disease of . xiii 532
injurious custom among . . vi 21
liable to diseases of heart . vi 21
Mines, foul air in . . . . . xv 81
Mirabeau, his tortures from pericarditis ii 318
Miscellany, the Military, by Mr. Marshall . . . . . xxiii 163
Misdoings of medical authors . xxi 499
Misery, and disease, connexion between v 448
by governments, remarks on . xiv 452
Missionary society, medical, in China xiv 216
Missions, medical, to China . . iv 568
Mistletoe, mode of its vegetation . iv 18
Mitchell, Dr., on the refuse of New York . . . . . xxi 334
Mitivie, M., and his lunatic asylum xix 611
Mitral valve, action of . . . . v 102
case of disease of . . viii 279
Mitscherlich, his Elements of Chemistry . . . ii 188, vi 505
on acetate of lead . . . iv 208
the Materia Medica . . viii 353
the production of ether . ii 190
Mobility, nervous, singular case of xix 304
of eye, mode of preventing . vi 57
Modelling process of reparation . vii 426-30
Moisture in atmospheric air . xix 8, 11, 12
on walls of apartments . . xix 9
Mojsisovics, Dr., on fractures of the thigh . . . . xvi 508
on the cure of syphilis by iodine xix 516
Molecular actions, in pathology . vi 298
motions in cells, Mr. Addison on xviii 100
Molecules, organic, and atoms, essay on xi 137
Mole, on a variety of fleshy uterine vi 92
pregnancy, curious case of . viii 567
Moles, and hydatids, origin of uterine iv 98
a product of conception? . xiv 210
Dr. Montgomery on . . xiv 210
in relation to legal medicine . xiv 210
M. Lisfranc on . . . . xviii 18
M. Mahon on the existence of xiv 210
uterine, on the diagnosis of . iii 404
Dr. Davis on . . . iii 403
on the origin of . . iii 404
Mollison, Dr., on the pulmonic pulse iv 241
Mollities ossium, analysis of . . ix 389
cause of . . . . iv 153
nature of . . xii 158
on pathology of xx 100, 272
Mollusca, ganglions in . . . ix 101
nervous system of . . v 494, viii 507
structure of the shells of . xxi 76
Molluscum, case of . . . ix 221
of the eyelid . . . . xviii 420
Monachism, remarks on . . iv 57
Monads, Dr. Mayer's doctrine of . iii 467
Monads, microscopic views of . xi 137
Monastermo, the smallest animal form xi 137
Monastic life, effects of, on females xiv 389
Mondini's membrane of the pigment ii 352
Mongolian race of mankind . . xxiv 460
Mongolian, the original race of man xxiv 479
Monguls and Laplanders, relation of xviii 380
Monkshood powder licked up by a pony xxiii 495
Monk-surgeons, history of . . xiv 405
Monkeys, &c., Mr. Neville's experiments on . . . . . iii 191
use of laryngeal pouches of . xvi 380
Monoculi, human monsters like . viii 11
Monomania, acute . . . xx 21
conscientious, case of . . vii 12
homicidal . . x 139, xvi 83, 91, 109
cases of . . . . . xix 40
remarks on . . . xxiv 240
imitative . . . . . x 170
incendiarism and theft in . x 147
incendiary . . . . . xx 28
nature of delusion in . . xxiii 236
opinions on . . . . . vii 11
religious, case of . . . i 287
restraint in . . . . . x 137
seldom feigned . . . x 137
validity of deeds in . . x 137
Monomania ophthalmica, case of . xiv 548
Monomaniac, the suicidal . . xx 22
Monomaniacs, responsibility of . vii 14
Monro, Dr., his anatomy of the bladder, &c. . . . xiv 540
on the spinal ganglia . . ix 111
Monster, anencephalous, how produced viii 16
case of a double female . . viii 33, 34
double, case of . . . xiv 255
parasitic, curious contents of . xiv 64
wonderful, of Dr. Mayer . iii 468
Monsters by defect, accounts of two v 169-70
civil rights of . . . ix 46
compound . . . . viii 30
production of . . . viii 17
double, defect of power in . xii 386
facts relative to . . viii 12
from accidents . . xii 387
from excess of power xii 384, 388
not from double ova . xii 383
on fusion causing . xii 384, 388
origin of . . . xii 383
parasite . . . . viii 35
Professor Vrolik on . xii 374
error of M. St. Hilaire on . xii 384
generalization of . . . xii 381
heteradelph . . . . . xii 375
monocephalous . . . viii 34
named acephali . . . viii 33
anidiens . . . . viii 33
autositaires . . . viii 30, 33
autosites . . . viii 8, 30
celosomi . . . viii 31
omphalosites . . . viii 30
paracephali . . . viii 32
parasites . . . viii 30, 33

Monsters, of anterior duplicity . xii 377

of lateral duplicity . . . xii 378

single . . . . . . viii 29, 30

sycephalous . . . . . viii 34

triple-bodied . . . . . xii 381

triple, rarity of . . . viii 36

united inferiorly . . . xii 381

posteriorly . . . xii 381

superiorly . . . xii 381

Monstrosities, causes of . . viii 15

history of . . . . . viii 2

St. Hilaire on . . . viii 1, 36

three classes of . . . viii 4

Monstrosity, a peculiar . . . . x 254

a remarkable . . vi 381, xiii 527

a singular fœtal . . . x 255

by inclusion . . . . . viii 35

double, case of . . . xxiv 325

Montault, M., on typhus . . viii 428

Mont-d'Or, mineral waters of . xiv 350

Montfaucon, curriers' yards at . xi 14

voirie of, description of . . xi 14, 17

Montgomery, Dr., on cancer of uterus xiii 571

on pregnancy . . . xiv 207

signs of pregnancy . . iv 448

Month, relative sickness in each . xxiv 94

Montpellier, climate and school of . xii 160

Montrose lunatic asylum . . ix 144

Moon, influence of, on chronic delirium xx 22

on disease . . viii 481

Moore, Mr., on puerperal fever . ii 481

Moral aspects of medical life . . xxiii 467

disorder, insanity from . . xx 13

feeling, nature of a . . xxiii 236

feelings, mental instincts . xi 101

guilt, boundary of . . . xv 422

insanity, mere, remarks on . vii 6

sentiments, insanity of . . xv 468

training of idiots . . . xxiv 14

Morals, effect of malaria on . . xviii 504

influence of climate on . . xiv 452

Morality, and prudence . . . xxiv 227

and religion of medical men . xxi 438

effect of civilization on . . xviii 244

of medical men . . . x 219

Morand, Dr., on medicine and surgery xxiii 372

Morbid anatomy, deficiency of works on xxiii 64

Dr. Carswell's *plates of* . i 152

four species of . . i 118

its use in nosology . . i 28

Mr. Travers's remarks on ii 30

on pathology from . . iii 12

Sir C. Bell on . . vi 156

study of . . . . xxii 323

treatises on . . . . xvi 381

growths, Mr. Liston on . . xxii 374

origin of . . . . i 120

habits of body . . . xxiii 59

poisons, Dr. Williams on xiii 89, xvi 290

nature of . . . . v 354

products, Prof. Vogel on . xxii 329

solid . xxii 334

state, origin of the . . . xxi 145

Morbid states and morbific processes xxiii 53

Morbidity of mind, remarks on . xviii 472

Morbility and mortality, on tables of xxiv 105

Morbilli sine catarrho, false measles xxi 467

Morbus carbuncularis, three forms of xiii 41

cardiacus, Dr. Seidlitz on . i 259

cœruleus, Dr. Kluge on . . i 254

treatment of . . . viii 50

mucosus in Gottingen in 1761 xxiv 354

mulieris, notice of the . . iv 128

Moreau, Dr., his psychological studies xxiii 217

on midwifery . . . xix 416, 421

Morehead, Dr., on diseases of Bombay xxi 381

on dysentery at Bombay . xxiv 333

Morell, Rev. J. D., on phrenology, quoted . . . . . xxii 528

Morgagni, his immortal work . vi 295

his opinions on dysentery . xxiv 334

his own case of dysentery . xxiv 348

Morgan, Mr., his surgery . . iv 185

on cataract . . . . xvi 326

diseases of the eye . . viii 229

inflammation . . . x 532

ophthalmic cases by . . vi 74

Morgan *v.* Boys, case of, as to a will x 138

Morgue, corpses in the, fastened down ii 412

Morphia, acetate of, cheap mode of making xiii 81

in pertussis . . ii 178

use of, in insanity . ii 167

bimeconate of, Dr. Thomson on viii 276

Mr. Squire on . . viii 275

endermic use of . . . v 348

in pertussis . . . ii 178

in tetanus . . . v 230

external use of, in gout . . iv 536

inoculation of . . . iv 506

in arthritis . . . . ix 252

narcotism of, antidotes for . v 347

salts of . . . . xiii 81

Morphium, endermic use of . . vi 268

in cancer . . ii 517

use of, in hydrophobia . . vi 268

Morphology, a contribution to . xxiv 135

Dr. Carus on . . . v 85

Mortality, among M. Broussais' patients ii 5

and morbility, on tables of . xxiv 105

at Geneva lunatic asylum . i 498

at various hours of the day . xviii 175

comparative tables of . . iii 44, 45

discrepancies in tables of . ix 348

effect of civilization on . . xviii 245

force of, in infancy . . iii 46

in manhood . . . iii 46

in old age . . . iii 46

from bells, in Rio Janeiro . ii 234

from systems of physic . . x 423

in America, Dr. Dunglison on i 352

Britain, Mr. Chadwick on . xv 333

cities, and towns . . iii 50

cause of higher . . ix 359

city and country districts . ix 358

England, and Ireland, compared xviii 42

and other countries . xxi 3

Mortality in England, and Wales . xi 423

various causes of . xi 426-8

infantile, report on . . xx 549

influence of time of day on . xxiv 116

in 42 professions in Geneva . i 194

Ireland in ten years . . xviii 35

last and present century xiv 450, xxiv 96

London, and Manchester . xv 74

half-yearly . . . ix 357

Milan hospital . . . xxiv 379

occupations in Paris . . xiv 448

professions, statistics of . x 373

Prussia . . . . . viii 575

relation to climate . . iii 49

six English cities . . iii 50

law of, in all organized nature iii 46

laws of, remarkable fact in . xxii 215

less in protracted fevers . i 408

male and female, in England . xi 424

mean, in latitudes . . xiv 448

new bills of, for London . ix 582

of British troops abroad . . iii 49

different races and nations . iii 48

females in London . . xv 337

Glasgow, report on . . xviii 492

St. Petersburg . . . . x 582

rate of, at different ages . iii 44

less in females . . iii 47

Mr. Edmonds on the . iii 40

ratio of, remarks on . . vi 7

relative rate of . . . vi 6

seasonal in three cities . . xxiv 95

sexual, table of . . . iii 47

English tables of . . . ix 347

table of, for London x 289, 589, xi 35, 267, 545, xii 285, 575, xiii 267, 577.

New York, 1805 to 1836 vii 272

by Dr. Lombard . . i 194

tables of, of 850 medical men . i 202

urban and rural, in England . xi 428

various causes of . . . xxiv 113

Mortification, amputation in . . xi 319

blackness as a sign of . . xii 122

causes of . . . . . xi 318

contrasted cases of . . xi 319

diagnosis of . . . . . xi 319

divisions of . . . . . xi 317

Dr. Carswell on . . . i 144

fætor as a sign of . . . xii 122

from pressure on . . . xxii 171

general symptoms of . . xi 319

in old age . . . . . xiv 91

nature of, Mr. Jones on . . xviii 268

of the male genitals . . ix 571

of toes, bandaging in . . iv 259

Sir C. Bell on . . . vi 157

treatment of . . . . . xi 320

Morton, Dr., his crania Americana x 474, 485

Morton, Mr., his anatomy of perineum viii 244

on inguinal hernia . . . xii 511

on the groin, &c. . . . viii 542

Mortuary registers of Geneva . . i 194

Morulus, a disease of the cuticle . vi 334

Moscow, epidemic cholera in, 1831-2 iii 485

foundling-hospital of . . iv 276

French retreat from, remarks on i 347

hospitals in . . . . . iv 275

medical school of . . . iv 272

Moss, Carrageen, with chocolate . xii 87

Iceland, bread of . . . xiii 66

Irish or Carrageen . . xiii 65, xvii 21

Most, Dr., on medical jurisprudence vii 530

on nux vomica in dysentery . i 256

on state medicine . . . xv 225

Motard, Dr., on general hygiene xiv 446, 461

plan of his work on hygiene . xiv 447

Motherby, Dr., his Medical Dictionary i 177

Mother-marks, singular case of . xiv 561

Mothers, advice to, by Dr. Kilgour . i 358

education of . . . . xiv 527

hints to, by Dr. Bull . . v 215

Motion, correlation of, Mr. Grove on xxiii 247

in the animal organism . . xiv 497

lesions of voluntary . . xx 28

M. Valentin on . . . xi 301

of the heart, principle of . . i 439

organic, Baumgäertner on . xxiv 133

semi-voluntary . . . xvi 368

voluntary, regulated by cerebellum xxii 531

Motions, animal, changes accompanying xiv 499

involuntary, Johnstone on . i 386

Motive, absence of, in insanity . xvi 84

apparent absence of, in crimes xvi 84-5, 91

Motive action, on the term . . v 504

powers governing the will . xxiv 226

Motives, in actions, to be judged of . xxiv 230

in relation to laws . . . xxiv 230

Motor apparatus, structure of . . xxiv 130

nervous power, its direction . xvii 173

phenomena, observations on . xix 299

power, origin and action of . xvii 174

Mouatt, Dr., his Hindustani anatomy xxii 227

on Bengal medical returns . xxiii 440

on causes of Indian diseases . ix 564

Moulinié, M., on surgery . . xvi 266

Moulting, in insects, remarks on . xvi 209

on the process of . . . xvi 209

Mountains, lofty, cultivation on . i 345

Mouth, affections of mucous lining of xi 411

and jaws, tumours of . . iv 147

case of spasmodic distortion of . iv 134

commencement of digestion in . xxiii 393

exploration of the . . . vi 138

gangrene of, actual cautery in . x 585

in children . . xxiv 46

in infants . . iii 444

of animals, diseases of . . xxiv 82

operation for new . . . vii 409

plastic operations on . xix 402, xxi 303

Movements, consensual . . . xix 310

remarks on . . xvi 372

sources of . . xxii 505

remarkable distinction of . xxiv 201

various, of animals, Unzer on . xxiv 200

Moxa, anti-erethistic effect of . . iv 217

cases treated by . . . iv 217-19

Dr. Sadler on the use of . . iv 217

in rheumatism . . . xiv 581

its effect in febrile affections . iv 217

in painful affections . iv 217

its general effects . . . iv 217

mode of applying it . . iv 217

purely dynamic effect of . . iv 218

reparative effect of . . . iv 218

revulsive effect of . . . iv 218

Moxas below clavicle in phthisis . xxii 469

employed by Dr. Sadler . . iv 217

Mucilaginous principle of aliment . xvii 8, 13

Mucous diseases, chronic, tongue in iii 72

mineral waters in . . xiv 340

lining of mouth, affections of . xi 411

membrane, causes of disease from xx 341

in different diseases . . xi 404

inflamed, colour of . . v 428

of air-passages, on . . xxiv 505

of stomach . . . ix 565

on it becoming skin . . xii 428

peculiar powers of . . iii 14

membranes, alum in inflamed . xi 524

in ulcers of . xi 524

inflammatory products of . xxiv 120

in renal disease . . ii 226, 228

morbid anatomy of . . xi 401

motions of . . . xi 402

secretions of . . . xvii 442

structure of . . . xi 402

Mucus, acidified, digestive properties of iv 201

how distinguished from pus . iii 531

insoluble in acids . . . iv 202

microscopical examination of . vi 546

on secretion of protecting . iii 14

organic, of plants . . . ix 502

profuse secretion of, in infants xi 205

Mucus-corpuscle, non-existence of . xxii 23

Mudàrr, or asclepias gigantea, use of xii 425

Mugna, Dr., on clinical medicine . xxiv 360

Muguet, and aphthæ, distinction between ii 367

Dr. Naumann on . ii 367

treatment of . . ii 367

and enteritis, identity of . . vii 85

an exudation on epithelium . vii 84

causes of, in the Enfans Trouvés vii 85

frequency and fatality of . . vii 86

general course of . . . vii 83

in Paris and St. Petersburg . vii 83

its difference from aphtha . vii 82

mean duration of . . . vii 84

or milk-thrush . . . vii 82

nature and character of . . vii 85

situation of . . vii 83-4

treatment of . . . . vii 86

Mühry, Dr., on homœopathy in Germany . . . . . xxii 565

Mulberry-like excrescence in trachea xxii 468

Mulder, on the buffy coat of blood xvii 431

on Liebig . . . . . xxiv 273

on medicine in France, &c. . iii 447

Müller, Prof., his Archiv für Physiologie v 76

his doctrine of life . . . vii 172

on motor nerves . . xvi 183

his Elements of Physiology viii 159, xiii 468

Manual of Physiology . v 75

Physiology (second edit.) . ix 244

notice of his works . . v 75

on artificial digestion . . iv 201

formation of bone . . viii 286

Mr. Newport's discoveries . v 620

nervous system . . . v 293

osteoid tumours . . xix 537

structure of cancer . . x 235

secreting glands . . ix 533

sense of hearing . . viii 68, 70

teeth . . . . . viii 158-9

vision . . . . . x 2

Mueller, Dr., on the nervous system vi 394

Munk, Dr., on delirium tremens . v 600

on the action of digitalis . . xxiii 417

Munster, medical school of . . i 299

society at . . ii 581

Murder, by carbonic acid gas . . vi 241

by the insane . . . x 134

number of deaths by, in Ireland xviii 43

Murderer, horrible case of a German xxiv 235

Muriatic acid in the fever of growth ii 220

vapour in phthisis . . iv 212

Muriate of barytes in white swellings ii 567

of iron in softening . . ii 551

lime, in diseased ovary . iii 139

Murmur, the uterine, remarks on . v 596

Murmurs in the heart, causes of . v 426

in the living body . . . v 592

on the two respiratory . . ix 300

pharyngeal, buccal, and nasal . ix 306

Murphy, Dr., on mercury . . ix 237

on the first stage of labour . iv 398, 401

Murrain transmissible to man . xxii 36

Murray, Dr., on the influence of heat xiii 349

Murray, Sir J., his air-pump theory iii 479

on the air-pump . . . iii 478

Murray, Fanny, Sir C. Bell's case of xxiv 498

Muscardine of the silkworm . . ix 400

Muscæ volitantes, on the cause of v 43, vii 280

Dr. Mackenzie on . . xx 518

in cataract . . . xi 80

Muscle, action of electric current on xxiii 402

fatty degeneration of . . xiv 543

structure of, plates of . . i 326-7

Muscles, and blood-vessels, relation of x 354

and tendons, division of . . xiii 1

rupture of . . . x 355

anomalies of . . . . xviii 224

case of inflammation of the . iii 227

contractility of, after death . xxiv 279

division of, in spine disease . ix 554

dorsal, on division of the . xii 253

extensive section of . . xi 250

femoral, division of . . viii 400

force of contraction of . . xix 259

influence of nerves in developing ii 543

Mr. Mayo on the . . . v 102

Muscles, Mr. Ward on the power of . xii 180

new, account of . . . . xiv 356-7

numerical anomalies of . . viii 23

oblique, of the eye, action of . xi 521

observations on the . . xvi 421

of animal life, structure of . xiv 275

inspiration, Stromeyer on . v 133

marasmus of the . . xxi 372

organic life, structure of . xiv 274

the back, on division of . xxi 332

coccyx, Theile on . . xiv 377

face, expression by the . xviii 450

hand, division of . . xii 257

neck, cases of spasm of iii 263

spine, division of . . xii 182

tongue . . . xiv 377

spasm-affected, treatment of . viii 404

state of, in spinal curvature . iv 509

structure of . . . . xix 257

tone of the . . . . xix 306

topical relation of nerves to . x 352

the mind in relation to . . xix 575

Muscular action, Bernouilli's theory of i 384

ingenious theory of . xxiv 132

in relation to the blood . xvi 421

physiology of . . . xiv 276

waste of structure by . xiv 499-502

without sensation . . iii 38

actions, classes of . . . v 505

contractility, experiment on . xiv 277

nervous system in . . xiii 483

remarks on . xiii 164, 478, 483

contraction, on induced . . xxiii 407

current of electricity . . xxiii 396

fibre, development of . . ix 517

M. Valentin on . . xi 301

structure of . . . xxiv 128

fibres, attachment of to tendons xvii 252

formed in disease . . x 260

fibrilla, ultimate composition of xxi 481

irritability, Dr. Reid on . . xiii 226

Dr. Williams on . . xvii 475

in winter-sleep . . xxiv 409

M. Longet on . . xiii 226

motion, Hunter on causes of . v 528

motions, on voluntary . . v 491

power, sudden loss of, case of. xi 506

retraction, syphilitic . . xiii 543

sense, in the deaf and dumb . xvii 242

of Sir C. Bell, on the . xxii 510

spasm, cured by myotomy . xiii 22

strength of the insane . . xxii 15

structure, on renewal of . . xvi 214

system, changes in . . . xvii 444

Dr. Wilson on . . xvi 418

of animals . . . v 488

predisposing to disease . xx 345

source of its tone . . iii 37

tone of the . . . xvi 168

tissue, action and growth of . xxi 548

development of . . xxiv 129

diversity of . . . xiii 164

report on . . . xvii 252

Muscular tissue, structure of . . xiv 273

Musculo-cardiac function, Dr. Wardrop's v 146

Musculus risorius of Santorini . xiv 376

Museum, Army Medical . . vii 225

British, Dr. Marx on the . xv 21

drawings from Army Medical . vii 224

Dublin, Catalogue of . . xi 513

formation of John Hunter's iv 80, 81, 83

Hunterian, Catalogue of the . xv 195

Dr. Marx on the . . xv 22

Hunter's, and Mr. Pitt . . iv 86

and Sir Everard Home . iv 86

of Blumenbach at Göttingen . i 500

Dr. W. Hunter, cost of the iv 191

Materia Medica in Edinburgh i 307

Soemmering at Giessen . xiv 374

St. Bartholomew's, Catalogue of xxiii 249

Mushrooms, used as food . . xvii 21

Music, Pythagoras' philosophy of . xviii 170

Musical murmurs . . . viii 456, 461

Musk, and ammoniacum, in tympanites ii 552

dissection of a dog poisoned by i 245

endermic use of . . . v 350

injected into the veins of a dog i 244

in palpitation of heart . . iv 399

symptoms in a dog poisoned by i 245

when indicated in typhus . xxii 473

Mussel, ciliary motions in gills of the i 509

Mustard applications, cold water for i 570

error in making . i 570

Mutatio sexus, origin of the fables of vi 76

MUTTER, Dr., his edition of Liston's

Lectures . . . . xxii 379

his operation on the nose vii 402, xxii 381

on club-foot . . . . xi 513

deformity from burns . xviii 525

plastic operations . . xix 396

Mutton, diseased, case of poisoning by xviii 332

Mydriasis, and amaurosis, different . v 43

case of idiopathic . . . ix 259

Myelitis infantum . . . . xiii 248

Myodynamometer of Valentin . xix 259

Myopia, cure of, by operation . xiii 238

apparatus for cure of . . x 567

cure of by myotomy . . xii 553

division of muscles for . . xii 253

lenses proper for . . . xvi 362

letter of M. Guerin on . . xii 554

Myopodiorthoticon . . . x 567

Myotomy, and tenotomy, history of xvi 66

rapid progress of . . xiii 23

in spinal curvature . . xii 283

Myriapod, cephalic part of head of xxii 503

Myriapoda, circulating system in . xx 496

on the growth of the . . xx 490

Mr. Newport on the . . xx 487

head of, Mr. Newport on . xx 493

structure of the . . . xx 489

Mysteries of nature, observation on xxiv 288

Mystery, the philosophy of . . xii 519

Mysticism, in German medicine, remarks on . . . . iii 450

medical, remarks on . . xxi 450

NAEGELE, Dr. F. C., on midwifery xix 58
NAEGELE, Dr. H. F., on midwifery xix 58
on obliquely contracted pelvis xi 167
on obstetric auscultation . viii 365
Nævi, acetum lythargyri in . . xv 239
materni, vaccination in . . x 26
subcutaneous, destruction of . xxii 171
treatment of, by injection . iii 265
the use of injections in . . xi 76
various modes of treating . xxi 290
Nævus, case of ligature of carotid for vi 233
cases of . . . . . xix 540
convulsion from injecting . v 613
fatal case of . . . . . v 613
Mr. Tyrrell's treatment of . xi 76
new mode of operating for . iii 557
treatment of . . . . . viii 53
NAGLE, Dr., on the stethoscope . v 596
Nails, crookedness of, in hectic fever xii 95
curved form of . . . ix 548
minute structure of . ix 510, xiv 266
rasped human, vomiting from xv 68, 73
state of, in fracture of limbs xii 548
structure of, report on . . xix 568
Nakra nasa, or nose disease of India iii 353
Name, remarks on an illustrious . iv 76
Names, changing of, strictures on . v 43
of medicines, danger from
changes of . . . . . xviii 561
table of new and old iv 116
Nantes, medical meetings on syphilis in v 1, 28
Naples, statistics of cholera in 1836 v 260
Naphtha, alleged cures of phthisis by xvi 490
use of, in phthisis . . . xix 146
wondrous effects of, in phthisis xvi 493
NAPOLEON, remarks on the disease of xix 27
the emperor, case of . . ix 478
Narcotic poisons, report on . . xviii 557
Narcotico-irritant poisons, report on xviii 562
Narcotics, endermic action of . v 346
observations on . . . . xvi 190
their use in insanity . . xxii 55
with astringents . . . xvi 200
mercurials . . . xvi 200
purgatives . . . xvi 200
Narcotine for quinine in ague . viii 263
Narcotism, a sort of, in scarlatina . ii 178
Nard, oil of, Dr. Forsyth's account of xvi 357
NARDO, Dr., on cantharides . . ii 548
Nares, new instrument for plugging iv 231
Nasal and olfactory nerves, connexion of xiv 381
bones, openings in, on closing xxi 298
catheter, mode of using . . iii 450
of Gensoul described . iii 449
duct, absence and formation of xii 541
obstructed, treatment of . xi 78
reopening the . . . xxi 311
style or tube for . . x 29
exudations, structure of . . xxiv 120
fossæ, closure of . . . xiv 223
polypi, formation of . . xxiv 121
tubes, extraction of . . x 29
NASMYTH, Mr., on the teeth viii 159, 168,
ix 443, xii 491
Nassau, on endermic bronchocele in xx 168
topography and statistics of xx 169
NASSE, Professor, on nervous fibres x 553
on milk . . . . . xi 228
reflex motions . . . x 257
secondary abscess . . iii 502
NASSE, Drs., their physiological researches iv 414
Natchez Indians, skulls of . . x 482
Nates, gangrene of, generally fatal . ii 29
presentation of, in labour . xix 70
National education, Mr. Combe on xxiii 254
Nations, brains, &c. of, Mr. Combe on x 485
of men, cranial divisions of . xviii 373
on the health and strength of xix 140
Natural course of disease always
interfered with . . . xxi 252
cure of diseases, on the . . xxiii 585
neglect of . xxiii 592
history, cabinet of, at Cairo . vi 592
education of idiots in . xxiv 13
minute researches in . xx 507
of animals . . . xix 556
of disease, mode of obtaining xxi 525
of Man, Dr. Prichard on xxiv 49, 441
of the mammalia . . xxi 496
on the study of . . ix 426
progress of, in Egypt . vi 592
utility of, to the mind . xxiv 218
philosophers of Greece . . xvii 458
philosophy, Dr. Bird's . . ix 529
elements of . . . xvii 523
value of . . . . ix 427
surgeon of Hunter . . . xxiii 588
Nature, and art, in curing diseases i 191, xxiii 594
and therapeutics, in disease . i 413
baneful practice of . . . xxii 559
cure of diseases by . . xxi 252
Dr. Symonds on the word . xxiii 599
kingdoms of, analogies of . xix 175
never to be thwarted . . xxi 520
observation of, Dr. A. Combe on xxi 509
in disease xxi 505, xxiii 257
on co-operation with . . xxi 512
exclusive trust in . . xxii 557
living in accordance with xviii 237, 245
regard to, in practice . xxiii 470
watching, in disease . . xxi 515
power of, in curing diseases . ii 270
Sir J. Herschel on the study of ix 428
the curative agent in disease xxi 519
NAUMANN, Professor, on congestion, &c. iii 209
Nausea, distension of stomach in . xv 63
repressed by creasote . . ii 169
Naval commands, mortality in . xi 194
statistics of . . . xi 192, 193
force in China, diseases of . xxii 180
hospitals, lectures at . . xviii 221
reports necessarily imperfect . xi 183
surgeon, letter to editor from a xxiii 265
surgeons, advantages of . . xxiii 107
advice to . . . xviii 221
returns required from . xi 182
Navel-string, case of long untied . v 583
strange treatment of . ix 532

Navigation, remarks on the art of . vi 104

Navy and army, comparative health of xvii 313

mortality in xvii 316, 328

relative health of xv 1, 13, 17, 18

various diseases in, relative prevalence of xvii 316, 328

Navy, British, diet in . . . xvii 48

cleaning of decks in . . xi 186

condition of the sailor in . xvii 313

dysentery in, from putrid water xvii 26

former bad condition of . . xi 187

greatly improved health of . xv 10

great mortality formerly in . xi 188

improved diet in . . . xi 185

health of, causes of . . xv 11

improvement in water for . xi 185

late great improvements in . xi 187, 189

libraries in . . . . xi 187

medical statistics of . . xi 182

mode and effect of watching in xi 185

mortality in, in South America xv 6

present healthy state of . . xi 188

rarity of consumption in . xv 13

report on the health of . . xv 1

returns of sick and hurt in . xi 184

sleeping accommodations in . xi 186

ventilation in . . . xi 187

Nebular hypothesis . . . xix 156

Necessities, physical, of man . . xiv 447

Neck, case of dislocation of . . vi 543

ecchymosis from ligature on . v 176

encysted tumours in . . xv 155

Necker, l'Hôpital, account of . i 281

Necremia, Dr. Williams on . . xvii 501

Necrosis, and caries, Lisfranc on . xv 30

in bone, cause of frequency of xii 157

internal, Dr. Klencke on . xvi 504

M. Petrequin on . . . . xx 138

of bone, nature of . . . xii 157

of cartilages of the larynx . v 432

of sternum, preparation of . xv 399

regeneration of bone . . xii 157

Needles in the parietes of the heart xx 224

Negro, and orang-outang, brain of . viii 381

and Egyptian races, report on xvii 274

brain and cord of, measurement of viii 379, 382

brain of, discussion on . . v 585

weight of . . viii 377, 382

capabilities of . . . viii 374

comparative size of brain of . viii 374

cuticle of, cause of colour of . xix 567

face of, observations on . . xviii 446

hair of, different from wool . xxiv 71

his dissimilarity to the ape . xix 180

leg-bones and arms of . . xxiv 80

preparation of muscles of . xv 203

race, Coptic origin of . . xxiv 444

races in Africa, remarks on . xxiv 446

rete mucosum of . . . vi 574

size and weight of his brain . iv 529

tribes in Africa . . . viii 383

women, period of puberty in . xiv 574

Negroes, female, healthier than male iv 262

form of their skulls . . xviii 384

in West Indies, diseases of . iv 262

mortality of . iv 261

statistics of . iv 261

mortality of . . . . vi 150

Pelagian, account of . . xxiv 469

Negroland, oceanic, or Kelænonesia xxiv 470

Neill, Dr., on the arteries and nerves xxii 231

Neison, Mr., his vital statistics . xxi 1, 4

on Mr. Chadwick's views . xviii 199

Neisser, Dr., on meningeal inflammation xxii 384

Neligan, Dr., on materia medica . xvii 529

Neonatorum œdema, cause of . vii 93

Nephritic attack from use of rhubarb xvii 25

complaints, on pains of back in xi 359

Nephritis, acute, four forms of . viii 154

idiopathic, rarity of . xi 358

anatomical characters of. . viii 155

arthritic . . . . x 303

diagnosis of . . . x 304

causes of . . . . viii 151

chronic, effects of . . . viii 155

mineral acids in . . viii 156

urinary sediment in . viii 154

coagulable urine in simple . viii 153

diagnosis of . . . . viii 152

Dr. Prout on rheumatic . . xi 359

error of Dr. Prout on . . xi 358

four species of . . viii 150-51

from morbid poisons . . x 302

in puerperal women . . viii 156

in typhus . . . . x 303

leeches to perineum in . . viii 156

predisposition to, in old age . viii 151

prevention of. . . . viii 156

purulent urine in . . . viii 154

rarity of, in children . . viii 151

rarely from cantharides . . viii 151

Rayer's valuable cases of . viii 157

state of urine in simple . . viii 153

symptomatology of . . viii 152

symptoms of chronic . . viii 154

treatment of . . . viii 156, xi 359

Nephrotomy, M. Rayer on . . xv 475

Nero, baths of, effects of moist air in xix 267

Nerve, abductor, function of . . xi 283

accessory and first cervical . i 550

report on . . . xxii 284

-actions, three kinds of . . xxiv 208

Unzer on . . . xxiv 196

auditory . . . . xix 579

functions of . . . xi 287

papillæ of . . . vi 420

case of anæsthesia of fifth . vii 544

division of, heat after . . viii 197

facial . . . . vi 200, xix 580

Bellingeri on . . . ix 143

function of . . . xi 286

-fibres, central ends of . . xix 573

structure of . xix 572, xxii 271-73

terminations of . . xxii 273

Nerve, fifth . . . . . . xix 579

Dr. Alcock on . . iii 257

first cervical, origin of . . i 550

glosso-pharyngeal . . . xix 580

functions of . . xi 287

hypoglossal, functions of . xi 290

report on . . xxii 284

inferior laryngeal . . . . xix 584

infraorbital, Sir C. Bell's error on xxii 492

oculo-motor, functions of . xi 282

of alimentary instinct . . vii 547

olfactory . . . . . xix 579

functions of . . . xi 279

optic . . . . . . xix 579

functions of . . . xi 280

pathetic, motor action of . xi 283

phrenic, functions of . . xi 294

pneumogastric, functions of . xi 517

seventh, portio dura of . . ix 131

spinal accessory, functions of . xi 294

sympathetic, Dr. Proctor on . xviii 528

motor functions of . . xi 295, 304

Mr. Swan on . . . xxiii 582

on tying the . . . iii 155

organic fibres of . . vii 501

origin of. .. xi 297, 304, xiii 344

sensory functions of . xi 297

source of its power . . ii 133

structure of . . . vi 411

third, physiology of . . xxii 280

trigeminus, irritation of . . xi 230

wounded, remarkable case of . vi 271

Nerves, action of electric current on xxiii 403

material ideas on . xxiv 193

on muscles . . xiv 499

poisons through . iii 16

anastomoses of . . . xii 236

anatomy and physiology of . xix 579

anæsthesiæ of . . . xvii 419

and arteries, outlines of . . xxii 231

and blood, Dr. Heidler on . xxii 218

and vessels, formation of . xxiii 139

apology for . . . . . xix 240

auditory, structure of . . vi 411

biotic properties of . . xxiv 187

causes of inflammation of . iii 24

morbid excitement of iii 23

central termination of . . xiv 282

cerebral, continuity of with others ii 135

motor influences of . xii 239

Mr. Grainger on v 498, 499, 593

of the frog . . . vii 237

origin and continuation of xviii 141

cerebro-spinal, structure of . vi 404

cervical, motor influences of . xii 244

chief action of, in animals . viii 510

ciliary, morbid affections of . xviii 50

circulation in . . . vi 407

cold and heat in relation to . iii 15

concreto-generalia of . . xi 299

contents of the tubules of . vi 406

correspondence of, Mr. Mayo on v 534

cutaneous, distribution of . xviii 143

Nerves, cutaneous network of . vi 416

determining the functions of . xiii 479

*diagram* of, four sets of . . xvii 182

disorders of, two kinds of . vi 146

dissection of, use of spirit in . xi 495

division of, Dieffenbach on . xxi 332

Dr. Berres on the structure of v 219

Dr. Hall's classification of . vi 217

Dr. Ley on forcible distension of iii 23

Dr. Reid on the 8th pair of v 308, viii 264

on the functions of . . ii 600

effects of section of various . iii 7, 14

eighth, influence of, on digestion viii 478

electrical currents in . . xii 245

electricity as the motive agent of viii 406

experiments on, by Flourens . i 244

on divided . . . viii 407

on the roots of . . ix 547

extremities of . . v 296, 299

fibrous distinction of the . v 293

fifth and seventh, Sir C. Bell on ix 128

fifth pair, Bellingeri on . . ix 142

functions of . . xi 284

J. Hunter on . . xv 213

various authors on . ix 112

functions of . . . . . xi 277

eighth pair of . vi 574

roots of . . xii 239

ganglia of, discovery of new . xv 232

homæsthetic, and sensible . xxiv 189

hyperceneses of . . . xvii 420

hyperæsthesiæ of . . . xvii 408

incident excitor, and reflex motor xxiv 205

incident motor, Dr. Hall on . xvii 175

inflammation in relation to . iii 14, 15

inflammation of, Dr. Ley on . iii 23

influence of, on circulation . iii 16

injury and disease of, Dr. Ley on iii 20, 22

intimate structure of . . vi 404

law of Bell on roots of . . xi 278

locomotive, of animals . . viii 510

loop-like terminations of v 295-6, vi 414, 417

metal between divided . . viii 407

minute elementary structure of v 294

structure of ix 517, xiv 277, xvii 263

authors on . v 293

motor, physiology of . . xxii 279

Mr. Mayo on root of fifth pair . v 534

Mr. Swan's illustrations of . ii 192

M. Valentin on . . . xiv 380

muscular, distribution of . . xviii 143

no peripheral termination of . vi 417

of cornea . . . . . ix 548

eye, Van der Kolk on . . xviii 48

facial expression . . iv 212

gravid uterus, dissections of . xi 494

lungs, experiment on . . xxi 553

male genitals, report on . xxii 288

motion and sensation, Sir C. Bell on . . . . . ii 215

palate and tongue, Burdach on vi 418

sensation and motion, Galen on i 382

smell and taste . . . xviii 143

Nerves of taste, discussion on . v 110

Dr. Alcock on . . iii 259

the will, in animals . . viii 510

uterus and mammæ, connexion of x 513

uterus, Dr. Lee on . . xi 493

Tiedemann's views of xi 494

vessels, sympathies of . . ix 409

vision, affections of . . xviii 414

olfactory, structure of . . vi 411

optic, structure of . . . vi 411

termination of . . vi 419

organic fibres of . . . vii 501

organization of tubules of . vi 405

origin of fifth and seventh . iv 212

origin of, M. Foville on . . xviii 439

the roots of . . v 495-8

pains from disease of sensitive . iii 22

particular, report on . . xvii 266

peculiar reflex functions of . vii 547

peripheral, affections from . xvi 255

symmetry of . . . xi 298

terminations of . xiv 281, xix 574

petrous, Dr. Bidder on . . vi 204

phrenic, effects of tying . . iii 155

physiology of . . . v 296

Prof. Müller on . vi 424

plates of the cerebro-spinal . iv 497

pneumogastric, and accessory . xix 581

offices of . . . iii 155

on tying . . . . iii 154

report on . . . xxii 183

primitive fibres of . v 294, vii 501, xi 299

prolongation of the tubules of . vi 405

recurrent, effect of section of . iii 29

redness of, after death . . iii 23

reflex action of . . . xvii 264

Unzer on . . xxiv 195

regeneration of . . . xvi 503

relation of, to skin, brain, &c. . xxiv 131

relations of . . . . ii 215

repair and union of . . xvii 263

respiratory, of animals . . viii 510

reunion of . . . . xvi 369

divided . . . vi 170

roots of, Grainger and Bell on . v 495-7

Mr. Mayo on . . v 486

sensual, are incident-excitor . xix 302

sentient and motor, structure of vi 408

sequelæ of inflammation of . iii 24

Sir C. Bell on . . . ix 124

size of, in the Negro . . viii 381

some effects from wounds of . vi 271

spinal, accessory functions of . xi 517

and cranial, Sir C. Bell on ix 127

Dr. Macartney on the roots of ii 135

report on . . . xvii 268

stretched over glands, case of . iii 24

structure of . . . vii 500, xxiv 131

*plates of* . . i 327

substance of, tubular . . vi 404

sympathetic, aud vagus, in animals x 509

connexions of . . . xvii 383

report on . . . xvii 268

Nerves, sympathies of, Whytt on . xix 99
terminal loop of . . . xiv 281
terminations of . . . vi 414
disputes on . . . vi 418
in muscles . . . vi 414
in the skin . . . vi 415
their share in sensation . . vi 217
topical relation of, to muscles . x 352
trunks and branches of, consentient iii 21
tumours of, treatment of . . xxi 292
two sets of fibrils in . . xxiv 193
ultimate distribution of . . v 295
Valentin on regeneration of . xi 304
varicose tubules of . . . vi 407
where sensible of injuries . iii 20
Nervi vagi, vomiting after division of xii 528
Nervous action, diagram of . . xvii 174
doctrine of Whytt, &c., on iii 6
laws of, M. Flourens on xvi 365
M. Longet on . . xiii 226
speciality of . . xvi 365
transmission of . . vi 215
without the brain . . xxiv 202
affections, acetate of morphia in ix 252
Dr. Canstatt on . xiii 343, 346
on local . . . . xii 121
local, from cerebral disease iv 135
of young women . . vii 146
Sir B. Brodie on . . iv 132
and capillary systems, relation of iii 12, 13, 16
urinary system, connexion of xvi 215
vascular systems, connexion of i 393-6
apparatus, vascular system of . ix 99
atmosphere of Kluge . . vii 320
centres, anatomy and physiology of . . . xix 577, xxii 274
centres, composition of . . xvii 442
inflammation of . . xvii 528
influence of . . . xvii 264
circle in animals . . . viii 510
deafness, two forms of . . iii 98
debility, Dr. Heidler on . . xiv 337
depression, alcohol in . . xxiv 529
disease, intense, our ignorance of nature of . . iii 31
mode of observing . . xiv 55
diseases, deaths from, in England ix 353
debility in . . . iii 223
Dr. Bene on . . . i 23
Dr. Chiappa on . . i 252
Dr. M. Hall on . . xii 200
Dr. Rowe on . . . xviii 229
Dr. Stokes on . . ix 492
mineral waters in . xiv 336, 338
obscurity in . . . xvii 126
of women . . . xii 60, 75
points of inquiry in . . xiv 59
Prof. Romberg on . . xvii 407
reflections on some . . iii 328
spasm in . . . xiv 57
theory of . . . xx 220
disorder, from vitiated blood . iii 326
disorders, irritation in . . xiv 57

Nervous disorders, pressure in . xiv 57

energy, Dr. Ley on defective . iii 26

excitability, creosote in . . ii 169

exhaustion and depression . xviii 479

fever, effect of season on . . xxiv 110

of Huxham, nature of . xvii 362

fibres, alterations of . . x 553

course of . . . xiv 280

gray, of Müller, denied . xi 304

independent sympathetic . xiv 296

longitudinal, course of . xviii 134

vegetative . . . xviii 143

fibrils, distribution of . . xvii 384

fluid, or vital spirits, Unzer on . xxiv 185

force and actions, Dr. Todd on . xxi 453

not electrical . . . xxiii 407

functions, on alteration of . iii 327

ganglia, structure of . . xiv 283

ganglion-globules . . . xiv 282

headache, aconite in . . x 584

remarks on . . . xiii 147

impressions, centric and peripheral, action of . . xxiv 201

diffusion of . . . xix 307

transmission of . . iii 21

influence, central, what . . iii 209

Mr. Travers on . . iii 2

on a vivifying . . . iii 9

peripheric, what . . iii 209

injury, a remarkable effect of . iii 11

irritability, from brain and heart xxii 425

M. Gintrac on . . xxi 417

on puerperal . . . iv 398

irritation, timorous . . xv 421

matter, gray and white . . v 500, 501

gray, function of . . xiii 478

structure of . . xiv 282

of brain, Dr. Gurlt on . vi 514

principles regarding . iv 417

properties of gray . . v 492, 593

the office of white . . v 493

mobility, characters of . . iii 224

singular case of . . xix 304

morbid action, metastasis of . xii 76

movements, co-ordinated . xvii 393

ophthalmia, Lisfranc on . . xv 32

pain of left side . . . iv 132

pains, and spasms, stop in sleep iv 134

illustrations of . . iv 133, 135

parts most liable to . iv 134

pathology, present limits of . iii 31

physiology, late researches in . v 532

power, and electricity, analogy of xxiii 408

Dr. Clark on . . . i 391

powers, evidence of design in . xvii 176

principle and vital action, Mr. Travers on ii 2

properties, speciality of . . xvi 366

spinal bodies . . . . xviii 134

stitches, remarks on . . i 24

structure, renewal of . . xvi 214

substance, nucleated cells . ix 518

substances, arrangement of . xiv 283

superexcitement, affections from xxi 417

Nervous, sympathies, Dr. Henle on ix 404
explained . . . xvii 393
system, action of, on the blood xxi 146
ancient notions of . . ix 98, 107
arrangement of . . xiii 481
a source of organic disease i 459
cells and fibres of . . xviii 140
changes in . . . . v 334
comparative anatomy of iii 491, xiii 480
controlling influence in . iii 10
development of . iii 490, xix 592
difficulty respecting . xviii 146
discoveries in . . . ix 96, 104
diseases of . . ix 492, xxi 273
in animals . . xxiv 89
in England . . xi 424
Dr. Bennet on . . xii 99
Dr. Carpenter on . . viii 506
Dr. M. Hall on iii 1, v 486, ix 580, xvi 158
Dr. Philip on the physiology of ii 131
Dr. Steward on . . xxiv 580
Dr. Verity on . . v 540
dynamics of, Unzer's . xxiv 181
effects of injuries of iii 5, 10, xviii 214
shock on . . . xiv 57
exact sense of the term . ii 135
excito-motor the . . . ix 101
exclusive diseases of parts of xiv 56
experiments on . . xvii 185
first trace of . . . i 389
functional division of . xvii 173
functions of . . . ix 99
general anatomy of . xix 572
physiology of xviii 144, xix 575
illustrations of iii 489, viii 226, x 508
in all animals . . . viii 510
connexion with heat . xi 395
inflammation . . xviii 69, 71
insects, Newport on . xx 487
the lowest animals . v 489
its agency on muscles . xvi 419
its separate functions . i 393
its sexual influences . xii 65
mechanical distinctness of xxii 493
minute anatomy of . . xviii 140
structure of . . ii 129
modifications of . . v 493
M. Bauzin on . . . xii 521
M. Burggraeve on . . xxiii 140
M. Flourens on . . xvi 364
Mr. Clark's anatomy of . iii 203
Mr. Mayo on . . . xiv 525
Mr. Newport on . . xvii 171
Mr. Owen on . . . xv 212
new theory of . . xviii 232
of females . . . xii 60, 75
office of each part of, in locomotion . . xvi 366
of the articulata . . xvii 181-2
invertebrata . . viii 506
lower animals . . xix 556
mollusca . . . viii 507

Nervous system, pathological query on iii 327

pathology of . . iii 2, 301, xi 452

plurality of functions in . ix 116

predisposing to disease . xx 345

present knowledge of . v 486

Prof. Müller on . . v 293

report on . . xvi 263, xix 572

sensori-volitional, the . ix 100

separate powers of parts of xvii 173

simplest idea of a . . xiii 479

Sir C. Bell's views on . i 393

subordination of functions of xvi 367

syllabus of lectures on . i 203-4

sympathetic . . . ix 103

remarks on . . ii 133

true office of . . . iii 9-10

two structures in . . ix 99

unity of . . . . xvi 367

various treatises on . xvii 379

volume on . . . xiv 380

systems, modifications of . iv 30

temperament . . . xv 426

Dr. Lévy on . . . xix 54

texture, functions of . . xiii 478

tissue, chemical elements of . xiv 519

Dr. Carpenter on . . xiii 165

tremor of children . . . vii 583

cases of . . xx 556

vesicular matter, queries on . xxi 456

Nervousness, constitutional, theory of iv 476

Nervo-skeleton, remarks on the . xxiii 474

Nervus accessorius Willisii . . xviii 136

Nettle, juice of, in menorrhagia . xx 539

Neuræmia, and neuræmic diseases . xix 100

in relation to insanity . . xix 102

Neuræmic diseases, diagnosis of . xix 101

etiology of . . xix 101

treatment in . . xix 102

Neuralgia, aconite in . . . xx 467

aconitine and veratria in . ii 500

actual cautery in . . . xiii 158

acupuncture in . . . v 276

acute, treatment of . . iii 263

affection of the hair in . . xiii 147

after amputation, cured . . ix 251

and rheumatism, diagnosis of . xiv 412

belladonna in . . . xiii 157

between the toes . . . xix 561

cardiac, treatment of . . xxiv 41

cæliac, or "the spasms" . xvii 416

causes of . . . . xiii 141

cervico-brachial . xiii 149, xvii 412

cervico-occipital . . . xiii 149

character of . . . xiii 138

cherry-laurel water in . . i 263

ciliary, treatment of . . xvii 411

course and duration of . . xiii 140

creosote in . . . ii 169, vi 270

crural . . . . xvii 412

case of . . . . xiii 153

cured by acupuncture . . ix 251

turpentine . . . xii 432

definition of . . . . xiii 137

Neuralgia, description of . . xviii 57

diagnosis of . . . . xiii 142

and treatment of . . iii 262-3

dorso-intercostal . . . xiii 150

Dr. Allnatt on . . . xvi 511

Dr. Hall on, criticised . . xiii 158

Dr. Hunt on . . . . xviii 56

Dr. Rowland on . . . vii 529

effect of civilization on . . xviii 250

essential nature of . . . xiii 141

facial, Sir B. Brodie on . . xxii 170

femoro-popliteal—sciatica . xiii 154

from anemia . . . . xviii 63

disease of the brain . xviii 65

disordered liver . . xviii 62

dyspepsia . . . xviii 59

lead . . . . . x 447

mechanical causes . . xviii 65

spinal irritation . . xviii 63

uterine disease . . xviii 64

incision in . . . . . ix 258

intercostal, mistakes in . . xiii 152

intermittent and remittent . xviii 67

lancinating pains in . . xiii 140

lauro-cerasus in . . . i 263

lumbo-abdominal . . . xiii 152

mercury in . . . . vi 573

M. Valleix on . . xiii 136, 158

narcotics in . . . . xiii 157

nature of . . . . . v 276

of cervix uteri, case of . . xiii 153

breast, remarks on . . xxii 158

fifth nerve . . . . xvii 410

heart, M. Bouillaud on . ii 337

inferior dental nerve . . ix 258

portio dura, question on . xiii 148

pneumogastric . . . xvii 414

spinal cord . . . xvii 417

testis . . . . . xvii 83

oil of turpentine in . . xiii 157

pain from pressure in . xiii 138, 146

pains in . . . . . xiii 138

periodical, quinine or arsenic in xiii 157

periodicity in . . . xiii 147

purgative plan in . . . xiii 156

rarity of spasm in . . . xiii 159

relative prevalence of . . vi 14

remedies for . . . . xxi 332

rheumatic, diagnosis of . . xiv 412

salivation in cases of . . xiii 147

sciatic, diagnosis of . . xvii 411

treatment of . . . xvii 411

seat of . . . . . xiii 138

sesquioxide of iron in . . xiii 157

strychnine in . . . . x 588

successive blisters in . . xiii 157

termination of . . . xiii 141

treatment of . . . . xiii 156

trifacial . . . . xiii 143

diagnosis of . . . xiii 147

two kinds of . . . . vi 146

cerebralis, or hemicrania . xvii 418

facialis, operation for . . xii 283

Neuralgiæ, cutaneous . . . . xvii 410

various causes of . . . . xvii 409

Neuralgic and inflammatory pains . iii 25

habit, treatment in . . . xviii 59

pains, diagnosis of . . . . iv 134

in the insane . . . . iv 133

on the seat of some . . . iv 133

Neural, cranial, arches in the cod, *figs.* of . . . . . xxiii 485-6

nasal, arch in the cod, *fig.* of . xxiii 486

Neurasthenia typhica . . . . viii 442

Neurilemma of the nerves . . . vi 404

structure of the . . . . xiv 277

Neurological knowledge, results of xvii 380

researches by Dr. Bidder . vi 204

Neuromata, on pain from . . . iii 25

Neuronosi, Dr. Lanza on . . . xxiii 55

Neuroparalytic senile inflammation xvii 107

Neuropathy, minor degrees of . iv 472

or nervousness, Dr. Gully on . iv 471

Neurophlogistic senile inflammation xvii 107

Neurosis of the stomach . . . xviii 208

Neuroses, functional divisions of . xvii 407

of the heart, M. Bouillaud on ii 336

predisposing causes of . . xiii 344

prolegomena on the . . . xix 555

scientific consideration of . xvii 407

spinal, treatise on . . . . xxi 280

the motor . . . . . . xvii 419

Neurotic affections, classification of xiii 344

crises of . . . . xiii 345

Dr. Canstatt on . . xiii 343

extension of . . . xiii 345

metallic salts in . xiii 346

prognosis of . . . xiii 346

progression of . . xiii 346

rhythm of . . . xiii 345

Neutral salts, effects of, on the blood xiv 144

NEVERMANN, on rupture of the uterus x 486, 493

NEVILLE, Mr., on insanity . . . iii 189

appendage to . . . . iii 190

New Annals of Medical Science, German i 219

NEWBIGGING, Dr., on auscultation xiv 173, 180

Newborn infants, on food and physic to ii 383

pemphigus in . . . vii 94

New editions, on pretended . . . xvi 511

New Englanders, Mr. Combe on the xv 57

NEWMAN, Mr., his Zoologist . xxiv 436

NEWNHAM, Mr., on human magnetism xix 429

his surgical report . . . . viii 546

work recommended . . . iii 198

on disorders of literary men . iii 197

the body and mind . . xv 413, 429

works of . . . . . . xv 413

NEWPORT, Mr., his discoveries iii 291, v 620

his researches in physiology . xx 487

importance of his researches . xvii 181

Muller on his discoveries . . v 620

on insect temperature . . . iv 527

insects . . . . . . ix 533

the brain of myriapods . xxii 500

nervous system, &c. . xvii 171

New South Wales, diseases at Sydney v 261

New York bakers, practices of . v 411

College of Physicians, &c. of ii 585

Hospital, state of . . vii 1, 53

New Zealanders, description of . xxii 468

NICHOLLS, Mr., on hydriodate of potash . . . . . . viii 597

NICOLAI, Dr., on medical jurisprudence xvi 68

Nicotian criminals, valuable hint to xiii 63

NIEUWENHUYS, Dr., his report of fevers, &c. . . . . . iii 161

on Dutch medicine . . . vi 275

Niger expedition, Dr. M'William in xvi 259, 261

fever on the . . . . xvi 259

history of the . . . . xvi 259

list of the . . . . xvi 260

mortality table of . . xvi 261

fever, causes of . . . . xvi 263

nature of the . . . . xvi 261

sequences of the . . xvi 263

waters of the, on gas in the . xvi 263

Night-blindness, epidemic . . . xii 538

-study, Dr. Gregory of Edinburgh on i 342

-sweats, hectic, treatment of . xix 23

in phthisis, remarks on . xxii 87

Nile, dead-houses of the . . . xix 48

Nine-day fits, mortality from in Dublin, 1782 . . . . . . ii 97

Nipple, and areola, in pregnancy . iv 452

attachment of the . . . . x 113

colour of the female . . . x 112

composition of the . . . . x 112

cuticle of the . . . . . x 112

cutis of the . . . . . . x 112

erection of the . . . . x 113

form of the female . . . . x 112

mamilla, position of . . . x 111

nerves of the . . . . . . x 113

openings of lactiferous tubes of x 113

principal arteries of . . . x 113

sore, leaden shield in . . . xiv 582

tincture of catechu in . xiv 582

supernumerary, cases of . . xx 538

use of eversion of the . . . x 108

Nipples, absorbents of the . . . x 113

blood-vessels of the . . . x 113

diagnosis of pregnancy by the . ii 403

sore, M. Méricourt on . . . xxii 156

supernumerary, cases of . . xxiii 91

Nisus formativus of Blumenbach . v 78

Nitras argenti, in gonorrhœa in women . . . . . . iv 258-9

Nitrate of silver applied to uterus . i 271

case of discoloration from ii 156

Dr. Hooker's use of . vii 265

Dr. R. Thomson on . vi 581

effects of, on stomach . xx 38

in blenorrhagia . . i 271

chronic cystitis . . vi 565

hemorrhoids . . xiii 62

laryngeal disease . . xxiv 497

white swellings . . xii 550

its internal use . . iv 116

mixture, for infants . . xx 362

Nitrate of silver, blue skin from . iv 116
preservation of . . xiv 557
skin affection from . . ii 156
use of, in chilblains . xiii 62
gonorrhœa . xiii 61
thrush . xxiv 432, 435
utility of . . . xiii 61
of soda, as an antiphlogistic . xix 241
Nitre, operation of, in pneumonia . xx 81
use of in acute rheumatism . xii 247
in detecting arsenic . xiii 194
Nitric acid, in phagedenic ulceration xi 321
test for, remark on . . xx 327
Nitrogen, as an element of food . xvii 3
assimilation of by plants . xi 440
favours putrefaction . . ii 396
in organic compounds . . xv 146
soil, source of . . . xvii 225
the system, opinions on . xvii 5
its influence on plants . . vi 552
quantity of, in food . . xxii 262
Nitrogenized food, on nutrition from xvii 38
vegetable principles . xiv 518, 519
Nitro-muriatic bath, case of cure by xiii 362
Dr. Lendrick on the . iv 254
ingredients of . . . iv 254
receipt for . . . xiii 362
Nitrous oxide gas, case of inhalation of v 114
Nivet, M., on female discharges . xiv 534
Noble, Mr., on phthisis in Manchester xv 310
on the health of factories . xvi 507
on the physiology of the brain xxii 488
Nocturnal emissions . . xv 351, 355
treatment of . xv 355-60
Nodes, venereal, treatment of . v 20
Noise in the ear, Dr. Kramer on . iii 98
Noli me tangere, iodine in . . viii 522
Nomenclature, a uniform statistical ix 351
medicinal, on the new . . iv 105
organo-pathological . . vi 128
Non-azotized substances in food . xiv 516
Non compos mentis, definitions of x 130
Non-mercurial treatment in syphilis i 17
Non-restraint in Bethlem . . xx 476
of lunatics, remarks on xix 297, 334, 598
Norfolk Hospital Museum . . xxi 283
North American Archives of Medical Science . . . . . i 230
North of England Medical Association ix 290
Norway, apothecaries in . . iv 546
hospitals in . . . . . iv 547
medical administration in . iv 548
degree in . . . iv 544
examinations in . . iv 542-43
instruction in . . . iv 542
literature of . . . iv 550
profession in, statistics of xv 282
midwives in . . . . iv 547
new medical bill for . . xix 188
on the language in use in . iv 541
population of, and crime in . xvii 88
prisons, Professor Holst on . xvii 88
scientific societies in . . iv 548
Norway, state of medicine in . . iv 541
statistics of lunacy in . . i 278
university in . . . iv 541, 545
vaccination in . . . iv 547
veterinary art in . . . iv 548
Norwegian leprosy, hereditary . xviii 351
cause of . . . xviii 351
Dr. Boeck on . . xviii 346
description of . xviii 347, 349
treatise on . . xiii 461
treatment of . . xviii 352
Nose, cure of obliquity of . . xiii 242
Dieffenbach's operation for new xi 197
-disease (nakra or nasa) of India iii 353
enlargement of, treatment of . xiv 249
muscles of . . . . . xiv 376
new, taken from the arm . xxi 302
on closing openings in bones of xxi 298
operation for a new v 245, vii 398, 400
for lessening large . . vii 403
for raising sunk . . vii 398
plastic operations on . xix 399, xxi 294
restoration of ala of, case of . xxii 381
of septum of . . . vii 403
of symmetry of . . xv 194
stony concretion in . . xi 238
sunk, elevation of . . . xxi 299
value of a new . . . xxi 302
Noses, improving turn-up . . vii 403
saddle-backed, improving . vii 403
Nosography, comparative . . xxiv 82
Nosological systems, origin of . xxiii 97
Nosology, and morbid anatomy, remarks on . . . iii 302
botanical . . . . . iii 452
cerebral, difficulties of . . iii 301
general views as to . . iii 302-3
in public returns, remarks on . xxiii 441
of Cullen, disadvantages of . xi 190
of morbid anatomy . . i 28
on discriminating facts in . xxiii 41
positive, Dr. Lanza's . . xxiii 37
remarks on . . . . . x 195
on statistical . . . ix 351
Schonlein's new system of . xxi 27
Nostalgia, effect of civilization on . xviii 249
Nostrils, on dilating and perforating xxi 302
use of leeches to lining of . ii 173
Notes and Recollections, Professional xxii 551
and Reflections, Dr. Holland's viii 482
Medico-clinical, Dr. Lippich's x 204
Notions and ideas, distinction of xxiv 11
Nottingham, Dr., on popliteal aneurism . . . . . xxiii 425
Nottingham Lunatic Asylum . . ix 145
Nourishment, reflections on . . ii 374
Novum Organum, the, applied to medicine i 404
Nucleated cells, anatomy of . . xxii 262
Nuclei, in cells of the chorda dorsalis ix 505
of the blood-corpuscles . . xiv 572
Nühn, Dr., on a gland in the tongue xxi 281
Numbers, harmony of . . . xviii 170
medical opinions from . . ix 344

Numbers, perfect, formation of . xviii 170

use of, in therapeutics . . ix 346

Numerical calculations, correct data in x 38

medicine, objections to . . xxiv 293

method, the, abuse of . . ix 459

a science . . . xii 13

Dr. Laycock on . . xxi 525

Dr. Symonds on . . x 462

example of . . . xii 15

high import of . . xii 16

illustration of . . . xiii 257

in cholera . . . xiii 257

in medicine . . . vi 2

in sciences . . . xii 5

M. Gavarret on . . xii 1, 13

M. Louis on . . . i 404

objections to . . v 157-60, xii 16

obstacles to . . . xxiii 106

remarks on . . vi 42, xiii 472

rules for . . . xii 15

treatment by . . . xii 17

mode of studying diseases . i 168

or statistical method . . xxiv 291

Nunciati, M., his spiral suture of intestine . . . . . xxiii 25

Nunnely, Mr., his anatomy . . ix 538

on erysipelas . . xiii 373, 382

Nurse, a good, description of . xviii 158

Nurses, diet of, remarks on . . ii 221

diseased, milk of . . . xvii 25

examination of the milk of . xviii 155

external appearance of . . xviii 156

in asylums, dress of . . xxiv 161

milk of, may be too nutritious vi 185

quality of their milk explained vi 182

remarks on the food of . . ii 383

the dreadful curse of . . ix 532

Nursing, artificial . . . . x 523

conception during . . . xvi 231

sick, effects of civilization on . xviii 243

Nutriment, defective, ill effects of . xvii 43

passage of, into the blood . xxiv 276

Nutrition, and formation, of tissues xiii 165

and reproduction . . . vii 179

of parts . . . xi 304

and secretion, Dr. Todd on . iv 281

centres of, Mr. Goodsir on . xx 290

theory of . . . xxii 265

chemical theory of . . . xiv 302-8

deficient, symptoms from . viii 525

diseases of, Dr. Williams on . xvii 497

Dr. Carpenter on . xiii 484-5, xv 276

from dead tissues, Dr. Prout on xvi 485

from nitrogenized food . . xvii 38

in its chemical relations xix 563, xxii 261

Mr. Addison on . . . xx 121

nervous agency in . . . vii 179

physiology of . . . viii 549

process of . . . . . xviii 91

report on . . . . . xvii 260

sources of, Mr. Jeffreys on . xvii 154

structural relations of . . xix 565

use of lungs in . . . i 246

Nutrition, vital actions in . . xvii 187

Nutriment of vegetables . . iv 16

Nutritious material in the blood . xx 72

substances, transformations of xvii 260

Nutritive absorption, Dr. Carpenter on xv 268

process, in bee-hives . . xvi 482

nature of the . . . xviii 101

vegetables, scale of . . xiii 488

Nuts, nutritious properties of . . xvii 19

Nux vomica, action of . . . xvii 170

analysis of . . . ix 60

bark (Kuchila) . . xvi 357

detection of . . . xvi 80

Dr. Jahn's failures with . viii 426

Dr. Lombard on . . i 249

in diarrhea . . . i 256

in diseases of the heart . i 249

in dysentery, Dr. Geddings on i 255

formulæ of . . i 256

in incontinence of urine . ii 555

in stomach complaints . iv 244

inferences from effects of xvi 179, 183

its effects on animals . . i 249

mode of exhibiting . . iv 244

Nymphæ and labia, adhesion of . xxi 327

Nymphomania, Dr. Davis on . . iii 402

not caused by oophoritis . ii 528

Nystagmus bulbi, removed by myotomy xi 489

Nysten, his experiments on the breath i 243

on anæsthesia from ether . xxiii 550

pulmonary vapour . . i 242

the breath . . . i 243

watery vapour from the lungs i 242

Oats, nutritive degree of . . xvii 19
serratula arvensis noxious to . iv 130
O'Beirne, Dr., on strangulated hernia vi 558
on tetanus . . . . . ii 595
Obesity and leanness, Dr. Prout on xi 355
Obituary of Dr. John Sims . . vi 594
Obliquities of uterus, tedious labours from ii 526
O'Bryen, Dr., on chronic cystitis . vi 565
Obscene subjects, medical writings on xix 238
Observation, in its highest sense . xii 10
in physiology, remarks on . v 336
medical, former mode of . xii 15
Medical Society of . . v 154
of disease, M. Louis on . . v 157
of Nature in disease . . xxi 505
pathological, remarks on . vii 74
plan of, by Dr. Hall . . xiv 55
remarks on . v 322, 332, xxiii 104
school of, fault of the . . vii 353
Society of Medical . . xix 390
Observing faculties, education of the ix 414
Obstetric art, history of the . . xiv 80
auscultation . . . vii 230, viii 365
history of . . . i 86
cases, reports of . . . iv 369
diagnosis . . . . . i 85
enormity . . . . . vii 570
exploration, Dr. Hohl on . i 85

Obstetric exploration, Dr. Kilian on . i 85
medicine, Dr. Ramsbotham on . xix 235
stethoscope, of Dr. Hohl . i 87
Obstetrician's duties in lying-in room ii 524
Vademecum, by Dr. Ryan . ii 523
Obstruction of bowels, treatment of viii 496
Obturator, elastic, for holes in palate xxi 307
Occipital artery, ligature of . . xx 47
Occiput, soft, age subject to . . xvii 375
convulsions in . . xvii 375
Dr. West on . . . xvii 566
essential nature of . . xvii 376
morbid anatomy of . xvii 373, 375
mortality from . . xvii 376
not a rare disease . . xvii 371
of early childhood . . xvii 371
perforated spots in . . xvii 374
predisposing cause of . xvii 377
symptoms of . . . xvii 374
tetanic spasms in . . xvii 375
treatment of . . . xvii 378
Occupatio medici ad lectos ægrorum i 3
Occupations, as causes of phthisis . xxiii 438
Oceanic Negroland, or Kelænonesia xxiv 470
or Pelagian races of mankind . xxiv 466
Ockel, Dr., on the calendula . xxi 472
Octocephali monsters . . . . viii 9
Ocular inspection of the chest, its use i 480
Oculist's Vademecum, Mr. Walker's xvii 233
Odes, ballads, &c., titles for . . xix 125
Odontography, Owen on x 208, xx 376, xxi 62
Œdema, cerebral, M. Pinel on . xx 23
treatment of . . xx 38
in cardiac disease . . . . x 323
indurated, of infants . . xi 202
in pregnancy . . . xvii 540
in renal disease does not pit . x 323
of the glottis . . . . . v 431
anatomy of . . . xxi 413
diagnosis of . . . xviii 296
diseases preceding . . xxi 414
M. Valleix on . . . xxi 412
nature of . . . . xiii 537
symptoms of . . . . xxi 415
treatment of . xviii 297, xxi 416
of labia in labour, treatment of xix 426
Œdema neonatorum, cause of . vii 93
frequency of . . vii 93
mortality of . . vii 93
Œdème pulmonaire of Devergie . ii 410
Œnanthe crocata, cases of poisoning by xviii 563
poisonous properties of xxiv 438
Œnanthic ether . . . . . viii 330
Œsophagotomy, history of . . xvi 53
Œsophageal nerves in deglutition . xvii 178
Œsophagus, and stomach, digestion of xvi 310
cancer of . . . . . . xxii 295
case of fatal abscess of . . xiii 230
rupture of . . . . xviii 335
stricture of . . . . iii 73
cases of spasm of . . . . iii 73
digestive solution of . . xviii 335
dilatation of, case of . . xx 221
Œsophagus, diseased states of . . xi 412
nervous affections of . . iv 135
schirrhus of, cases of . . xxiv 46
spasmodic affection of . . iii 72
stricture of, Dr. Chelius on . ii 261
strictures of . . . . . vi 164
Œgophony, new explanation of . xix 113
remarks on . . . . . i 483
Oesterlen, Dr., on syphilis . v 1, 28
Officers, and soldiers, mortality of . viii 222
of health, in France . . vii 590
Officiers de santé in France . . i 495
Ogres, origin of the designation . xxiv 461
Ogston, Dr., on death from drowning iii 564
Ohio (U.S.), medical college of . ii 589
Oil, cod-fish, in scrofula . . ix 553
-glands of the skin, account of xxi 200
-globules, use of, in the system xiv 442
of bitter almonds, nature of . xi 134
poisoning by . xviii 560
of spikenard, or oil of nard . xvi 357
varnish to fracture apparatus . xii 544
Oils, essential, adulteration of . x 582
Oily foods, indigestibility of . . xvii 32
principle of aliment . . xvii 10, 14
seeds, nutritious properties of . xvii 19
Ointment of mezereon . . xiv 441
O'Keys, performances of the vii 348, 350
Old age, acute diseases of . . xvii 107
agrypnia of, remedies for xvii 110
alterations of the thorax in i 234
anatomy and physiology of xvii 101
and decrepitude, Dr. Smith on i 323
atheromatous arteries in . xxii 422
cerebral apoplexy in . xvii 110
changes of vascular system in xvii 102
condition of the lungs in . i 235
spine in . i 234
diseases of . vi 190, xvii 100
gouty diseases of . . xvii 114
example of vigour in . xvii 276
hyperemia of brain in . xvii 109
insidiousness of diseases in xvii 106
intermittent pulse, normal in xvii 107
jarring pulse in . . viii 504
medical treatment of . viii 503
medicines suitable to . xvii 106
unsuitable to . . xvii 106
partial anæsthesia of vagus in xvii 106
pneumonia of, description of xvii 112
pulse more frequent in . i 556
quotation from Cicero on i 343
regular pulse, febrile in . xvii 107
rejuvenescence in . . vi 80
Riverius' three periods of xvii 101
Sir A. Carlisle on . . vi 189
syncope in . . . . xvii 110
topical bleedings best in . xvii 107
venesection in, unsafe . xvii 107
vis medicatrix naturæ in . xvii 106
weakening effect of bleeding in vi 49
Oldham, Dr., on extra-uterine fœtation xxiii 425
on polypus uteri . . . xviii 344

Old, hospices for the, in Paris . i 281

maids, in relation to polypi . iv 372

man of the mountain, account of xxiii 218

man, the "soul life" of the . xvii 103

medical authors, on a taste for xv 28

men, medical conduct to . xxi 449

people, benefit of exercise to . xvii 106

dietetic advice to . . xvii 105

inflammation in . . xvii 107

temperature, in relation to xvii 105

persons, fainting fits in . . vi 190

pneumonia of . . iv 501

respiration and pulse of the . i 553

women, thoracic organs of . i 554

Oleaginous assimilation, morbid . xi 355

Olefiant gas and alcohol, relation . xi 134

Oleum jecoris aselli . . . xiii 197

nigrum, account of . . v 129

an Indian medicine . v 129

Olfactory nerve, account of the . xiv 381

lobes of the brain, remarks on xxi 458

Oligæmia or anæmia, treatment of . x 67

Olivary bodies, and facial nerves . iv 212

in animals . . . xxiv 575

Oliver, Dr. his physiology . vi 195, xiii 222

Oliver, Mr., his Student's Companion vi 202

Olivina and extract of olive, in ague xxi 212

Omental sacs in hernia, Mr. Hewett on xx 93

Omentum, case of protrusion of part of iii 159

tuberculated . iv 54

"expatriated," Dr. Parrish on iii 362

gangrenous, treatment of . iii 364

protruding from wound, case of xxi 421

the great, a diverticulum . xxiii 143

treatment of, in hernia . . iii 362

Omnibus-riding, disadvantages of . v 404

Onanism, as a cause of insanity . vii 29

physical proof of habits of . xxiii 508

Ononis spinosa, in rheumatism . x 558

Onychia maligna, new treatment of iii 266

Oophoritis, best detected through rectum ii 526

chronic, symptoms of . . ii 529

Dr. Löwenhardt on . . ii 526

periods most subject to . . ii 527

Ooteitis, rarefying, nature of . . ii 536

Opacity of cornea, treatment of . v 35

Operations, and symptoms, Galen on xiii 208

capital, mortality after . . xxiii 562

Mr. Cooper on . . xiv 132

results of . . . xv 240

delay to be avoided in . . v 457

during prostration . . . x 587

fatal, from internal disease, cases of ii 7, 8

forbidden by internal disease . ii 6

in America, statistics of . . xii 223

in the mesmeric state . . xix 434

remarks on performing . . v 417

rendered painless by ether . xxiii 310

rhinoplastic . . . . xxi 294

surgical, history of . . . xvi 46

on death after . . . xviii 333

on the jaws . . . xx 195

treatment after . . . xv 402

Operations, trivial, fatal results from ii 7

unprepared for, anecdotes of . vi 167

use of mesmerism in . . xxii 475

physical signs before . ii 7

Operative medicine, on the term . iv 195

surgery, Dieffenbach's . . xxi 285

Operatives, in manufacturing towns xiv 459

Operators, useful hints to . . v 457

Ophidians, spinal nerves of . . viii 227

Ophthalmia, acute, active treatment in xviii 405

artificial . . . . x 25

baryta in strumous . . ix 552

Belgian, nature of . . . ii 505

blisters over the eye in . . iii 450, 513

bloodletting in . . . ii 347

calomel and cotton in . . xx 463

opium in . . iii 150

catarrhal, contagious . . ii 504

diagnosis of . . . vii 220

Mr. Tyrrell on . . xi 61

treatment of . . . xviii 406

cause of its contagiousness . ii 504

cauterization of nose in . . xxiii 373

chronic, iodine in . . . viii 523

chronic scrofulous . . . xi 65

compound, Mr. Tyrrell on . xi 72

contagion of . . . . vi 197

Dr. Eble on the nature of . vi 197

Dr. Slade on . . . vii 217, 220

effect of civilization on . . xviii 251

Egyptian, Dr. Eble on . . x 3, 22

prize essay on . . . iii 288

epidemic, pathology of . . xx 82

exaltation of vision in . . xix 466

external use of calomel in . v 251

from mucus conveyed from vagina ii 503

from too great depletion . . xi 60

from severe injuries . . ii 268

gonorrhœal . . . . . x 25

in females . . . vii 220

three forms of . . . x 394

treatment of vii 220, x 395, xviii 408

in a school at Brühl . . ii 502-3

in Belgian army . . . vi 197

cause of . . ii 506

in Belgium . . . . ix 557

ravages of . . . ii 501-2

in Egypt, notice of . . . ii 501

in Russian army . . . ii 501

origin of . . ii 506

in soldiers, from dress . . iv 282

intermittent . . . . x 34

purulent, case of . . ii 507

lead collyria in . . . vii 219

liquid dropped into ear in . xviii 407

miasmatic . . . . x 32

mode of propagation of . . ii 503

Mr. Jeaffreson on . . . xviii 406

Mr. Lizars on . . . x 538

Mr. Middlemore on . . ii 346

nervous, Lisfranc on . . xv 32

of the modern Egyptians . . xviii 410

phlebitic . . . . . x 33

Ophthalmia, place for leeches in . vii 219

puerperal, Dr. Lee on . . xxiii 413

purulent, and gonorrhœa, analogy of ii 504

causes of . . . ii 503-4

corrosive sublimate lotion in ii 507

in infants . . . xi 62

Jüngken on . . . ii 501

mistura gonorrhoica in . ii 507

Mr. Tyrrell on . vii 155, xi 62

nitrate of silver in . . ii 507

of infants . . iii 443, xviii 410

treatment of ii 505-6, vii 156, xvii 234

Tscheterkin on . . ii 501

pustular, Mr. Tyrrell on . . xi 61

quality of secretion in . . ii 504, 506

rheumatic, treatment of . . xv 403

scrofulous, conium in . . viii 256

leeches in . . . vii 220

Mr. Tyrrell on . . xi 64

nose affected in . . xxiii 373

new light on . . . xviii 406

simple acute . . . . . xi 60

change of air in . . xi 60

chronic . . . xi 64

strumous, mercury in . . x 584

sympathetic . . . . . x 33

syphilitic . . . . . x 381

traumatic, Dr. Schindler on . ii 267

treatment of . . . . . vi 198

variolous . . . vii 220, x 32

remarks on . . . . xviii 411

ulcers on the cornea in . . ii 506

Ophthalmia neonatorum, blisters in vii 219

calomel in . . xiv 559

cause of . . x 393

treatment of . . x 394

Ophthalmiæ, study of the . . x 30

Ophthalmiatry, history of . . x 4

Ophthalmic diseases, bloodletting in xi 55

mercury in . . xi 57

Mr. Vines on . xiii 522

use of, to students ii 343

institutions, remarks on . . ii 342

medicine, treatises on . . x 1

Mr. T. W. Jones on . . xxiii 246

report of Prof. Rosas . . iii 517

surgery, Mr. Walker on . . xvii 233

remarks on . . . viii 229

therapeutics, Rognetta's . . xxi 159

Ophthalmitis interna, symptoms of . xviii 51

Van der Kolk on . xviii 48, 51

post-febrile . . . . xviii 192

Ophthalmological school of Vienna . x 5

Ophthalmology, Dr. Ammon's journal of iv 480

Dr. Rognetta on . . x 3, 21, xxi 158

history and progress of . . v 31

in Austria . . . . . v 31

Britain . . . . . x 7

England . . . . . v 32

Italy . . . . . . x 7

Switzerland . . . x 7

Journal of (German) . . ii 232

study of . . . . . x 4

Ophthalmoscopia . . . . x 23

Ophthalmoscopy, Dr. Marchetti on . v 31, 36

objective . . . . . v 37

subjective . . . . v 37

Opiate, warm, diaphoretic . . xx 444

Opiates, during labour, danger of . iv 403

Dr. Hamilton on iv 403

on dosing children with . . xxi 355

on poisoning infants by . . xviii 504

Opinion, changes of, remarks on . xv 426

change of a dominant . . xvii 472

Opium, abkaree, or Bengal bazaar . xvi 355

and emetic tartar, use of . . v 275

benefit from, in fever . . viii 439

Chinese investment . . xvi 355

detection of, Gusserow's method of v 213

in poisoning . ix 58-9

Opium-eaters, or Theriaki, in Turkey iv 394

Opium-eating, baneful effects of . iv 395

on its shortening life . xvi 355

Opium, effects of, on young children xx 332

on infants . . xviii 558

external use of, in croup . . iv 243

hydropathy a substitute for . xxii 453

in childhood, on the use of . xxiii 299

Indian, mode of preparing . xvi 354

in fever, disapproved of . . v 189

injurious effects of, in fever . vi 555

in perforation of intestines . ii 61

in pneumonia, M. Louis on . i 414

internal use of, in ulcers . . v 198

-liniment for croup, &c. . . iv 243

Mr. Houlton's new preparation of iv 540

Mr. Skey's mode of giving . v 199

Müller on the local action of . v 223

new preparations of . . iv 111

principles in . . . . ii 207

poisoning by, cured by hot water vii 62

treatment of . iv 142

properties and effects of . . v 197, 199

pure morphia in varieties of . xvi 355

quantity of, destructive of life . xviii 558

recovery from large doses of, without vomiting . . xviii 557

secondary asphyxia from . . xviii 557

sinking state after a dose of . xxi 106

-smoking, Dr. O'Shaughnessy on xvi 356

solubility of, in water . . xviii 558

test for, preferred by Mr. Taylor xxiii 416

therapeutic effects of . xxi 102, 105

use of, in hemorrhage . . xxi 107

in hernia . . . iii 358

in insanity . . . xxii 57

utility of, in small doses . . v 198

value of, in gangrenous inflammation . . . . . ii 30

with a diaphoretic, in labour . viii 473

with emetic tartar, in fever . xvi 238

large doses of emetic tartar i 420

Opisthotonos, pain in, not very great ii 595

OPPENHEIM, Dr., his Medical Journal i 546

on medicine in Turkey . . iv 380

syphilis . . . . v 2, 29

Optical delusion in a case of wry-neck xiii 3

delusions, case of . . . iv 380

Optic nerve, and sight, Mr. Grainger on v 513, 593

cerebral extremity of . v 593

nerves, divisions of the . . v 297, 299

origin of the . . . vii 282

termination of the . . vi 419

Oranges, diarrhœa cured by eating. xix 31

Orang-outang, and negro, brain of. viii 381

and pongo, identity of . x 251

on the extremities of the . xiii 474

Orations at the College of Physicians iv 189

Orbit, aneurism of the, case of . v 36

Orchitis, acute, secondary . . xvii 75

sequelæ of . xvii 75

advantages of compression in . ii 256

chronic . . . . . . xvii 80

yellow deposit in . . xvii 80

compression in . . . ii 253

consecutive, on mercury in . xvii 79

traces left by . . . xvii 76

cure of by pressure . . iii 516

effects of compression in . ii 254

gonorrhœal, from metastasis . xvii 78

origin of . . . xvii 77

in infants . . . . . xvii 79

mode of using compression in . ii 255

morbid anatomy of . . xvii 75

Mr. Curling on . . . xvii 74

M. Marc d'Espine on . . vi 59

new mode of treating . . xii 256

plaster for compression in . ii 255

syphilitic, character of . . xvii 81

dissection of . . . xvii 81

Ordure, animal, in towns, remarks on xxi 341

manure from, in France . . xi 17

Orfila, his experiments on the breath i 242

modified arsenical process of . xiii 194

on detecting arsenic . . xiii 183

hanging . . . . ix 266

poisoning . . . . xi 37

the breath . . . i 242

Organic actions, on the laws of . xv 137

agents, Dr. Prout on . . xxi 120

and chemical combination . xx 71

and inorganic kingdoms . . xiii 161

changes, pathological anatomy of vi 301

chemistry, Dr. Paine on . . xv 144

introduction to . . xviii 531

composition, unity of . . viii 4

development, laws of . . vii 176

disease after local injuries . ii 14

pre-existing, Mr. Travers on ii 6

remarks on the term . v 41

diseases, relative prevalence of vii 575

two groups of . . xxi 422

functions, and ganglionic system v 336

pathology of . . . iii 2

geological remains, Dr. Clark on i 392

life, in the planets . . . xix 163

the kingdom of . . xi 102

unfathomableness of . . xi 138

matter, Dr. W. Clark on . i 390

molecules and atoms, essay on xi 137

motion, Baumgaertner on . xxiv 133

Organic or sympathetic nervous fibres xiv 279

pathology, great error of the . vi 297

structure, diseases of . . vi 190

unity of . . . iv 478

surgery, Mr. Macilwain on . xx 127

vices . . . . . . xxiii 59

Organism and organization, Arnold on v 91

Organisms and cells, life of . . xx 71

Organizability or vitality, on latent xix 173

Organization and action, J. Hunter on v 48

anomalies of . . . . viii 1

azotized materials of . . xvi 283

medical, plan of . . . xxiii 105

new, of the profession . . xxi 531

of the profession . . . xix 215

progressive law of . . . xx 154

Organized beings, dispersion of . iii 367, 370

origin of . . . iii 367, 370

reproduction of . . xiii 166

bodies, peculiarities of . . vii 169

tissues, on development of . xiii 165

properties of . . . xiii 163

Organography, vegetable . . viii 243

Organo-pathological nomenclature. vi 128

Organs, affinity of for each other . viii 11

change of position in . . viii 21

formation of in the ovum . viii 7

gradual deterioration of . . ii 6

internal, situation of . . xix 103

law of balancement of . . viii 11

compensation of . . viii 11

not preexistent in the germ . viii 7

of animal life, report on. . xvii 261

similar position of . . . viii 11

size a measure of power in . ix 198

Orgasm, signification of the term . iii 210

venereal, cause of sensation in xix 511

Orifices of the heart, contraction of ii 326

diagnosis of narrowed . ii 328

dilatation of . . . ii 338

Original papers in British journals i 309, i 615

Origin of diseases . . . . xxi 145

Orthopædia, Dr. Brewer's analysis of xvi 352

operative, Dr. Stromeyer on . viii 384

subcutaneous . . . ix 227

discovery of . . . xiii 2

Orthopedic institution of Vienna . xix 369

operations, on the success of . xiii 2

science, remarks on the . . v 405

surgery of Dieffenbach . . xi 195

Orthopnœa in pericarditis, on . xxiv 556

relief in from mercury . . vi 388, 389

Osbaldiston, Sir A. Cooper's servant xvi 130

Osborne, Dr., on dropsies . . ii 222

on neuralgia . . . . v 276

thread setons . . . ix 578

Oscheoplastic, etymology of . . vii 412

operation . . . vii 412, xxi 312

Os cuboides, case of removal of . xv 400

Os frontis driven in, Mr. Travers's case of v 519

O'Shaughnessy, Dr., his Dispensatory . . . xvi 352, 359

on Indian hemp . . . . x 225

